# Aberrantly activated TAK1 links neuroinflammation and neuronal loss in Alzheimer's disease mouse models

**DOI:** 10.1242/jcs.260102

**Published:** 2023-03-13

**Authors:** Kazuhito Sai, Aoi Nakanishi, Kimberly M. Scofield, Debra A. Tokarz, Keith E. Linder, Todd J. Cohen, Jun Ninomiya-Tsuji

**Affiliations:** ^1^ Department of Biological Sciences, North Carolina State University, Raleigh, NC 27695-7633, USA; ^2^ Center for Human Health and the Environment, Department of Population Health and Pathobiology, College of Veterinary Medicine, North Carolina State University, Raleigh, NC 27607, USA; ^3^ Department of Neurology, University of North Carolina, Chapel Hill, NC 27599, USA

**Keywords:** Alzheimer's disease, Cell death, Inflammation, TAK1

## Abstract

Neuroinflammation is causally associated with Alzheimer's disease (AD) pathology. Reactive glia cells secrete various neurotoxic factors that impair neuronal homeostasis eventually leading to neuronal loss. Although the glial activation mechanism in AD has been relatively well studied, how it perturbs intraneuronal signaling, which ultimately leads to neuronal cell death, remains poorly understood. Here, we report that compound stimulation with the neurotoxic factors TNF and glutamate aberrantly activates neuronal TAK1 (also known as MAP3K7), which promotes the pathogenesis of AD in mouse models. Glutamate-induced Ca^2+^ influx shifts TNF signaling to hyper-activate TAK1 enzymatic activity through Ca^2+^/calmodulin-dependent protein kinase II, which leads to necroptotic cellular damage. Genetic ablation and pharmacological inhibition of TAK1 ameliorated AD-associated neuronal loss and cognitive impairment in the AD model mice. Our findings provide a molecular mechanism linking cytokines, Ca^2+^ signaling and neuronal necroptosis in AD.

## Teaser

**Summary:** TAK1, a protein kinase highly activated in the hippocampus of Alzheimer's disease model mice, kills neurons through necroptosis, leading to neuronal loss.

## INTRODUCTION

Neuroinflammation is associated with many neurodegenerative diseases and contributes to progressive neuronal loss in the brain of patients (Amor et al., 2010; Wyss-Coray and Mucke, 2002). In Alzheimer's disease (AD), inflammatory cytokines and deposition of amyloid plaques activate inflammatory responses in microglia. The reactive microglia secrete neurotoxic factors such as TNF, IL-1β, nitric oxide and glutamate that impair neuronal homeostasis and eventually cause neuronal cell death. Studies have shown that degenerating neurons further amplify inflammation through activating glial cells, and this feed-forward loop of neuroinflammation is a key aspect of AD progression (Block et al., 2007; Floden et al., 2005; Perry et al., 2010; Song and Colonna, 2018). Although the mechanisms by which glial cells are activated to subsequently secrete neurotoxic factors have been relatively well studied, how the neurotoxic factors cause perturbation of intracellular signaling in neurons and ultimately kills neurons remain poorly understood (Block et al., 2007; Jana et al., 2008).

TAK1, also known as mitogen activated protein kinase kinase kinase 7 (MAP3K7), is a key signaling hub for inflammation and cell death signals (Mihaly et al., 2014). TAK1 is a ubiquitously expressed protein, and is activated by various inflammatory stimuli, such as inflammatory cytokines, cellular stresses and pathogen invasion (Ninomiya-Tsuji et al., 1999; Orning et al., 2018; Takaesu et al., 2000). Once activated, TAK1 elicits cellular responses mainly through NF-κB and MAPKs, which result in transcriptional upregulation of inflammatory cytokines.

The enzymatic activity of TAK1 is tightly regulated by its interacting proteins, TAB1 and TAB2. TAB1 is required for activation of TAK1 upon stimulation, whereas TAB2 is implicated in both activation and deactivation processes of TAK1 (Broglie et al., 2010; Takaesu et al., 2000). We recently reported that TNF stimulation causes hyper-activation of TAK1 in cells lacking TAB2. This form of TAK1 activation leads to cellular damage through eliciting an inflammatory type of cell death known as necroptosis, indicating that perturbation of the regulation of TAK1 kinase activity results in undesired cellular damage (Geng et al., 2017; Jaco et al., 2017; Morioka et al., 2014). However, the physiological and pathological conditions that lead to aberrant activation of TAK1 have not yet been determined.

In the present study, we utilized AD model mice and primary neuron cultures to determine the possible involvement of TAK1 in neuronal cell death. Our results reveal that TAK1 is hyper-activated in the AD model mouse brain, which is causally associated with neuronal loss. We also demonstrate that neuronal TAK1 is hyper-activated via compound but not single stimulation with inflammatory cytokine TNF and Ca^2+^ influx, which leads to neuronal cell death. These findings contribute to a deeper understanding of the molecular mechanisms underlying AD pathology.

## RESULTS

### TAK1 is aberrantly activated in hippocampal neurons of Alzheimer's disease model mice

**Fig. 1.**
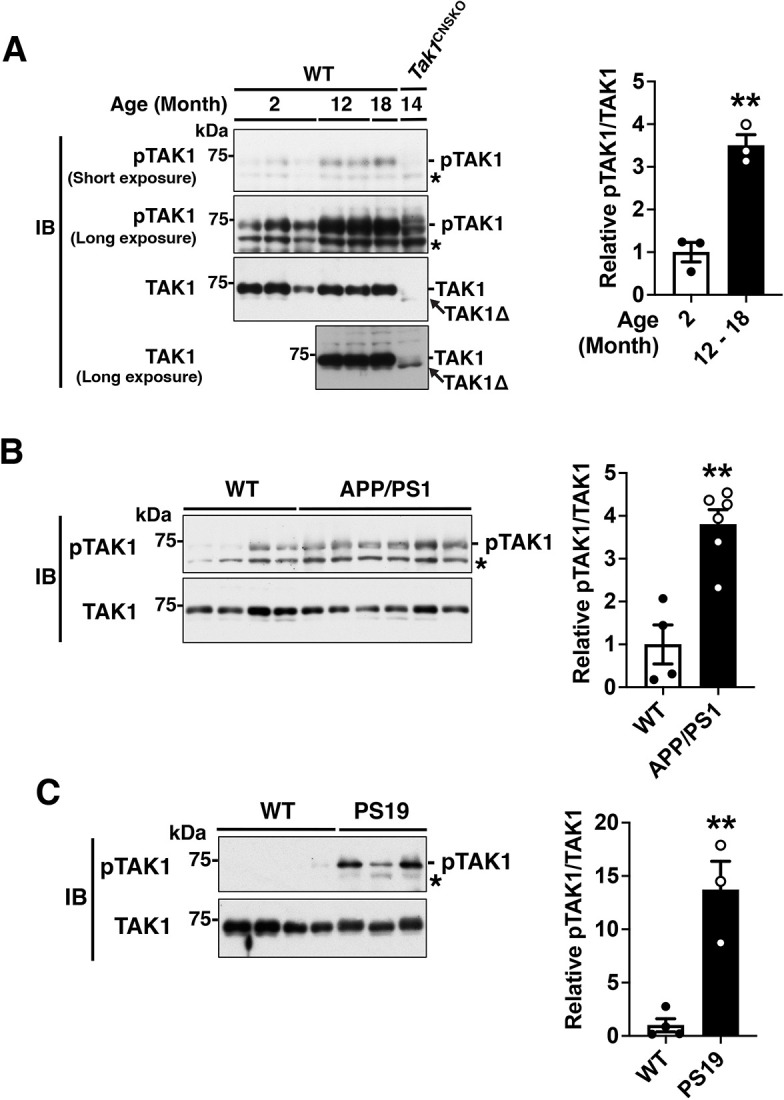
**Neuronal TAK1 activity is highly elevated in the hippocampus of aged and AD model mice.** (A) Left panels, hippocampal protein extracts from 2- to 18-month-old wild type (WT) and CNS-specific *Tak1* deficient (*Tak1*^flox/flox^ *Nestin-Cre*, denoted *Tak1*^CNSKO^) mice were analyzed by western blotting (IB) with the indicated antibodies. Each lane represents an individual animal. The activation status of TAK1 was determined by phosphorylation of TAK1 Thr-187 (pTAK1), which does not detect any inactivated TAK1 (Omori et al., 2012). Cre-driven in-frame deletion of the floxed *Tak1* exon2 generates truncated TAK1 (TAK1Δ). The bottom panel shows an longer exposure blotting of the same samples as the above panels but on the different membrane. *, non-specific band. Right graph, relative pTAK1/TAK1 levels of WT mice were quantified. (B,C) Left panels, hippocampal protein extracts from 12-month-old wild type (WT) and APP/PS1 (B) or PS19 (C) mice were analyzed by western blotting with the indicated antibodies. Each lane represents an individual animal. Activation status of TAK1 was determined by analyzing phosphorylation of TAK1 Thr-187 (pTAK1). *, non-specific band. For all graphs, all data points are shown, as well as mean±s.e.m. **P<0.01 (unpaired two-tailed Student's *t*-test).

We have previously shown that neuroinflammatory conditions induced by high-fat diet ingestion activates TAK1 in hypothalamic neurons, which contributes to deterioration of energy metabolisms by disrupting intracellular leptin signaling in mice (Sai et al., 2016). When investigating TAK1 activity in the several different brain regions, we found that the phosphorylation levels of TAK1 at Thr-187, which is correlated with the kinase catalytic activity of TAK1 (Kajino et al., 2006), were profoundly elevated in the hippocampus of aged mice compared with that of younger mice (Fig. 1A). *Nestin-Cre* is expressed in neural stem cell-derived cell types including neurons and astrocytes but not microglia or endothelial cells (Cho et al., 2013; Zimmerman et al., 1994), all of which express TAK1. In the *Nestin-Cre Tak1^flox/flox^* mice, Cre excises the floxed exon 2 of *Tak1*, which results in production of an in-frame truncated TAK1 protein (TAK1Δ) lacking the ATP-binding site only in neural lineage cells. TAK1Δ is catalytically inactive and unstable, and mice with this allele are phenotypically identical to TAK1-null mice (Sato et al., 2005; Shim et al., 2005). *Nestin-Cre* deletion of *Tak1* largely reduced intact TAK1 protein and activated TAK1 in the hippocampal lysates (Fig. 1A, far right lane), suggesting that TAK1 in neurons and astrocytes mainly contributes to the observed TAK1 activation. The low level of TAK1 activity observed with *Nestin-Cre* deletion is presumably derived from non-neural lineage cells such as microglia and endothelial cells. Elevated TAK1 activity was also seen in the prefrontal cortex but not in the cerebellum (Fig. S1). These findings prompted us to investigate the potential role of TAK1 in AD pathology, which is closely associated with age- and protein aggregation-triggered neuroinflammation in the hippocampus and the cortex (Blennow et al., 2006; Latta et al., 2015). To this end, we utilized two different AD mouse models – the APP/PS1 double transgenic mice expressing mutant human APP protein (Mo/HuAPP695swe) and mutant human presenilin 1 (PS1-dE9), which is a widely used model for amyloid deposition and behavioral analysis (Radde et al., 2006; Serneels et al., 2009; Webster et al., 2013), and the PS19 tauopathy mouse model, which is known to exhibit severe neuronal loss and memory deficits at relatively young age (6–12 months old) (Takeuchi et al., 2011; Yoshiyama et al., 2007). TAK1 activity was found to be highly elevated in the hippocampus of both APP/PS1 and PS19 AD model mice compared with the age-matched wild-type (WT) mice (Fig. 1B,C). These results demonstrate that TAK1 is aberrantly activated under AD-associated neuroinflammatory conditions in mice.

### Aberrantly activated TAK1 contributes to hippocampal neuronal loss in Alzheimer's disease

**Fig. 2.**
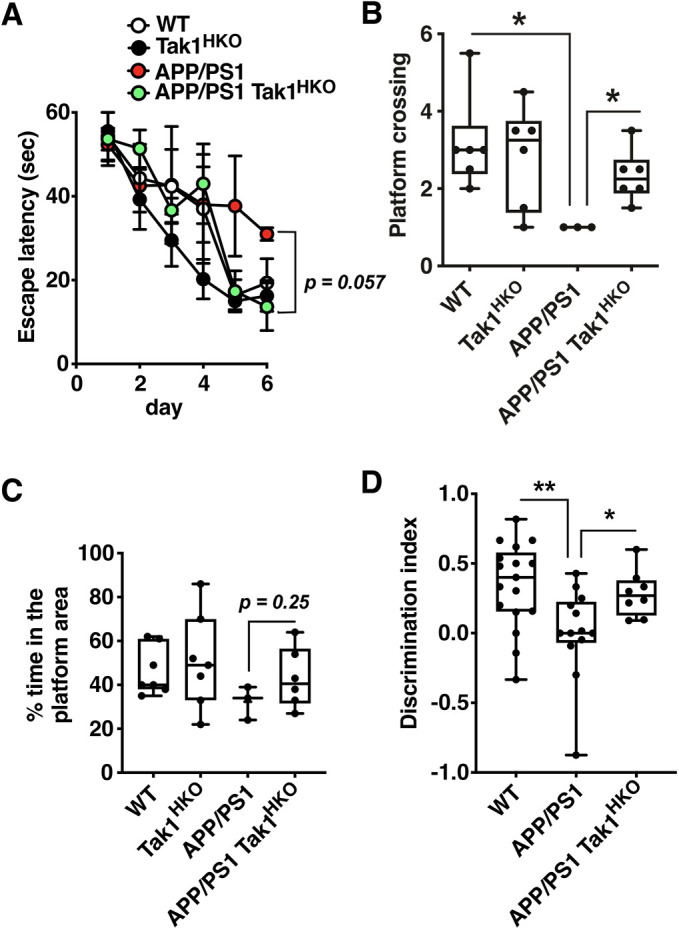
**Hippocampal neuron-specific deletion of *Tak1* alleviates cognitive decline of AD model mice.** The cognitive ability of 8–14-month-old wild type (WT) and APP/PS1 mice with or without hippocampal neuron-specific *Tak1* deficiency (*Tak1*^flox/flox^ *CaMKIIa-Cre*; denoted *Tak1*^HKO^) mice was tested by two commonly used tests, the Morris water maze (A–C) and novel object recognition tests (D). In the Morris water maze test, learn curves as the average latency (mean±s.e.m.) to locate the platform during the acquisition (days 1–6) (A), and all data points of percentage of time spent in the platform area (B) and times crossing on the platform area (C) in the probe trials at day 7 are shown. In the novel object recognition test, The Discrimination Index at the test session is shown (D). Water maze: WT, *n*=6; *Tak1*^HKO^, *n*=6; APP/PS1, *n*=3; APP/PS1 *Tak1*^HKO^, *n*=6; Novel object recognition tests: WT, *n*=17; APP/PS1, *n*=13; APP/PS1 *Tak1*^HKO^, *n*=8. *P<0.05; **P<0.01 (escape latency between APP/PS1 and APP/PS1 *Tak1*^HKO^ was determined by the simple linear regression test; other results were analyzed by the Student's *t*-test). On the box-and-whisker graphs, the box represents the 25–75th percentiles, and the median is indicated. The whiskers show the range.

In order to test whether aberrantly activated TAK1 in hippocampal neurons contributes to AD pathology, we generated AD model mice harboring a neuron-specific *Tak1* deletion. We utilized well-established tests of cognitive function, the Morris water maze (Morris, 1981) and the novel object recognition (Ennaceur and Delacour, 1988). As PS19 model exhibits hindlimb paralysis (Merchán-Rubira et al., 2019), which prevents accurate assessments in the behavior assays, we used the APP/PS1 model mice, which show cognitive impairment at 7 months old (Serneels et al., 2009). Consistent with the above studies, our APP/PS1 mice showed impaired spatial memory as assessed by the escape latency during the training sessions, the number of platform area crossings and the duration mice stayed in the platform area during the test session compared with WT littermates (Fig. 2A–C). We found that *CaMKIIα* (*Camk2a*)*-Cre*-driven hippocampal neuron-specific *Tak1* deficiency (Tak1^HKO^) (Dragatsis and Zeitlin, 2000; Wang et al., 2013) alleviated the impaired learning curve of APP/PS1 mice (Fig. 2A) and restored the number of platform area crossing (Fig. 2B). These mice also exhibited a trend towards higher percentages of time in the platform area compared with APP/PS1 mice (Fig. 2C). To confirm the results, we conducted additional a well-established cognitive test, novel object recognition (Ennaceur and Delacour, 1988). Whereas APP/PS1 mice were incapable of discriminating a new object from a familiar object (discrimination index≈0), APP/PS1 mice harboring hippocampal neuron-specific *Tak1* deletion exhibited improved scores (Fig. 2D). These data demonstrate that TAK1 contributes to the pathogenesis of AD in mouse models.

**Fig. 3.**
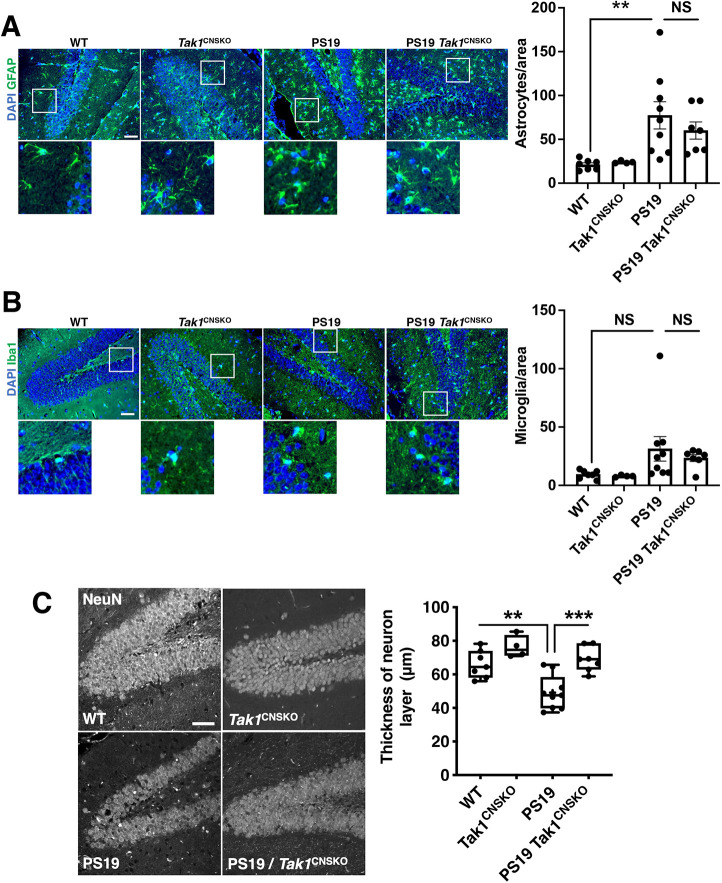
**CNS-specific deletion of *Tak1* blocks neuronal loss of AD model mice without altering neuroinflammation.** (A,B) Left panels, representative pictures of immunohistochemistry with anti-GFAP antibody (an astrocyte maker, A) or anti-Iba1 antibody (a microglia maker, B) conducted on hippocampal DG sections from 8- to 12-month-old wild-type (WT) and PS19 mice with or without CNS-specific *Tak1* deficiency (*Tak1*^flox/flox^ *Nestin-Cre*; denoted *Tak1*^CNSKO^). Scale bars: 50 μm. Right graphs, ruantification of the number of glia cells in an area of the DG. All data points are shown as well as mean±s.e.m. **P<0.01; NS, not significant (*P*≥0.05) (one-way ANOVA with Tukey's multiple comparisons test). (C) The thickness of dorsal DG neuron layers (visualized by NeuN staining) from 8- to 12-month-old wild-type (WT) and PS19 mice with or without CNS-specific *Tak1* deficiency was measured. On the box-and-whisker graphs, the box represents the 25–75th percentiles, and the median is indicated. The whiskers show the range; all data points are shown. WT, *n*=7; *Tak1*^CNSKO^, *n*=4; PS19, *n*=9; PS19 *Tak1*^CNSKO^, *n*=7. **P<0.01; ***P<0.001 (one-way ANOVA with Tukey's multiple comparisons test). Scale bars: 50 μm.

We next determined which pathological events in AD TAK1 is involved in. AD pathological events can be summarized as follows in the transgenic AD model mice: (1) pathogenic proteins form protein aggregates; (2) activation of glia cells, including microglia and astrocytes, which secrete various inflammatory cytokines and other neurotoxic factors, such as high concentrations of glutamate and nitric oxides; and (3) neurotoxic factors secreted by glia cells ultimately kill neurons (Block et al., 2007; Long and Holtzman, 2019). We first tested whether *Tak1* deficiency alters accumulation of protein aggregates in the hippocampus of AD model mice. Intracellular accumulation of phosphorylated tau proteins was observed in the hippocampus of PS19 mice, which was not seen in wild-type mice (Fig. S2A). We found that phosphorylated tau proteins were similarly accumulated in the hippocampus of PS19 mice harboring CNS-specific *Tak1* deletion (Fig. S2A). To quantify the levels of neurofibrillary tangles in these mice, we conducted western blotting using hippocampal extract. Neither total protein amount of tau nor phosphorylation levels of tau was altered by *Tak1* deficiency, indicating that TAK1 is not involved in the process of formation of pathogenic protein aggregates (Fig. S2B). Second, we examined involvement of neuronal TAK1 in neuroinflammation. As expected, the hippocampus of PS19 mice showed increased number of both microglia and astrocytes (Fig. 3A,B). It might be reasonable to assume that TAK1 participates in astrocyte- and/or neuron-driven neuroinflammation in AD model mice. However, the number of glia cells in the hippocampus was not observably altered in PS19 mice harboring CNS-specific *Tak1* deletion (Fig. 3A,B). We also examined the expression levels of inflammatory cytokines and chemokines in the hippocampus. We found that expression levels of TNF, IL-1β, C-C motif chemokine ligand 2 (CCL2) and C-C motif chemokine ligand 3 (CCL3) were markedly increased in the hippocampus of PS19 mice, whereas the IL-6 expression level was comparable to that in wild-type mice (Fig. S3). Consistent with the histology results above, CNS-specific *Tak1* deletion did not decrease the expression levels of those inflammatory genes. Taken together, these results demonstrate that the neuronal and astrocytic TAK1 pathway is not a major mediator of inflammation in the hippocampus of PS19 mice. Third, we examined whether hippocampal neuronal loss in the AD model mice was altered by *Tak1* deletion. PS19 mice are known to exhibit severe neuronal loss in the hippocampus as early as 8 months of age (Yoshiyama et al., 2007). Immunohistochemistry staining of a neuronal marker, neuronal nuclei (NeuN; also known as RBFOX3), showed that the number of neurons in the dentate gyrus (DG) of the hippocampus, measured as the thickness of the neuron layer, was less in PS19 mice compared with their wild-type counter parts (Fig. 3C). We found that CNS-specific *Tak1* deficiency markedly restored the thickness of neuron layer in the DG of the hippocampus (Fig. 3C). Collectively, these data demonstrate that TAK1 signaling in CNS mediates hippocampal neuronal loss downstream of accumulation of neurofibrillary tangles and neuroinflammation in the mouse models.

### TNF and glutamate-Ca^2+^ signaling cooperatively induce aberrant activation of TAK1

**Fig. 4.**
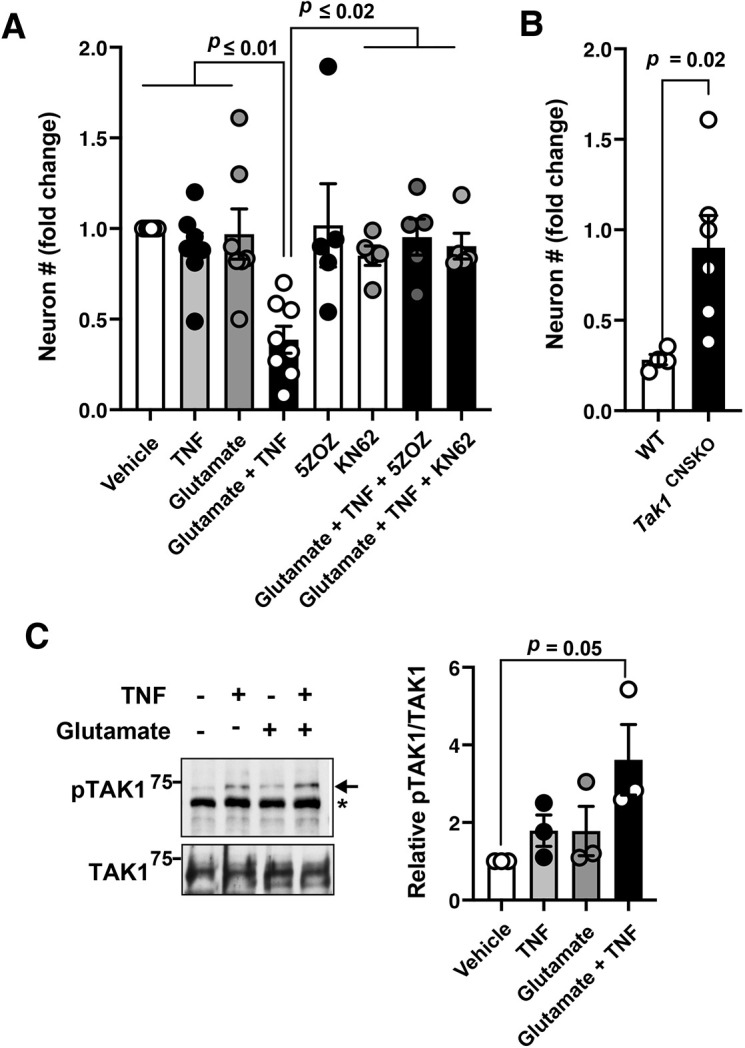
**Compound treatment with TNF and glutamate kills neurons through aberrant activation of TAK1.** (A) Wild-type mice embryonic neurons were treated with the indicated chemicals (TNF, 50 ng/ml; glutamate, 20 μM; 5ZOZ, 300 nM; KN-62, 10 μM) for 3 days. Neurons were fixed and stained with anti-TUJ1 antibody and DAPI. The number of TUJ1-positive neurons from three fields was counted. The graph shows fold changes of neuron number (relative to the vehicle-treated control). All data points from individual experiments using four different litters are shown as well as mean±s.e.m. *P*-values are shown (one-way ANOVA with Tukey's multiple comparisons test). Representative pictures are shown in Fig. S4. (B) Embryonic neurons from wild-type and CNS-specific *Tak1* deficient (*Tak1*^flox/flox^ *Nestin-Cre*; denoted *Tak1*^CNSKO^) mice were treated either with vehicle control, or 50 ng/ml TNF and 20 μM glutamate for 3 days. The number of TUJ1-positive neurons from three fields/treatment was counted. The graph shows fold change of neuron number (relative to the vehicle-treated control). WT, *n*=4; *Tak1*^CNSKO^, *n*=6. All data points and mean±s.e.m. are shown. *P*-value calculated by unpaired two-tailed Student's *t*-test. (C) Primary cortical neuron cultures were treated with 50 ng/ml TNF and 20 μM glutamate as indicated for 3 days. The activation status of TAK1 was determined by phosphorylation of TAK1 Thr-187 (pTAK1). *, non-specific band. Relative pTAK1/TAK1 levels were quantified (right graph). All data points and mean±s.e.m. are shown. *P*-value calculated by unpaired two-tailed Student's *t*-test.

We next sought to determine the molecular mechanism underlining hyper-activation of TAK1 in the hippocampus of aged and AD model mice. As chronic neuroinflammation is a hallmark of AD, one might think that inflammatory cytokines are the cause of aberrant activation of TAK1. However, cytokines alone only transiently activate TAK1, and this form of activation does not cause any cellular damage (Hanafusa et al., 1999; Morioka et al., 2014; Ninomiya-Tsuji et al., 1999). Consistent with this notion, none of the glia cell-derived factors solely cause neuronal cell death when treated at physiologically achievable concentrations (Floden et al., 2005). On the contrary, the combination of these neurotoxic factors, e.g. TNF and glutamate, can effectively induces neuronal cell death, although the underlying molecular mechanisms are not fully understood (Floden et al., 2005). We hypothesized that compound stimulation alters TAK1 activation kinetics, which leads to sustained and hyper-activation of TAK1 and ultimately to cell death. Consistent with an earlier study (Floden et al., 2005), co-treatment with TNF and glutamate effectively killed mouse primary cortical neurons, whereas individual treatments had minimal effects on viability of the neurons (Fig. 4A; Fig. S4). A pharmacological inhibitor of TAK1, 5Z-7oxozaenol (5ZOZ) (Ninomiya-Tsuji et al., 2003), blocked this cell death, suggesting that neuronal cell death induced by compound TNF and glutamate treatment is mediated by TAK1 (Fig. 4A). Consistent with this, *Tak1*-deficient primary cortical neurons were resistant to compound treatment with TNF and glutamate treatment (Fig. 4B). We also examined whether TNF and glutamate stimulation activates TAK1 in primary cortical neurons (Fig. 4C). Whereas TNF alone moderately activated TAK1, co-treatment with TNF and glutamate caused elevated TAK1 activity (Fig. 4C). This raises the possibility that glutamate-Ca^2+^ influx signaling might switch the canonical TNF–TAK1 pathway to the TAK1 hyper-activation pathway in neurons.

### TNF and the Ca^2+^-CaMKII axis cooperatively induce hyper-activation of TAK1 that leads to necroptotic cellular damage

**Fig. 5.**
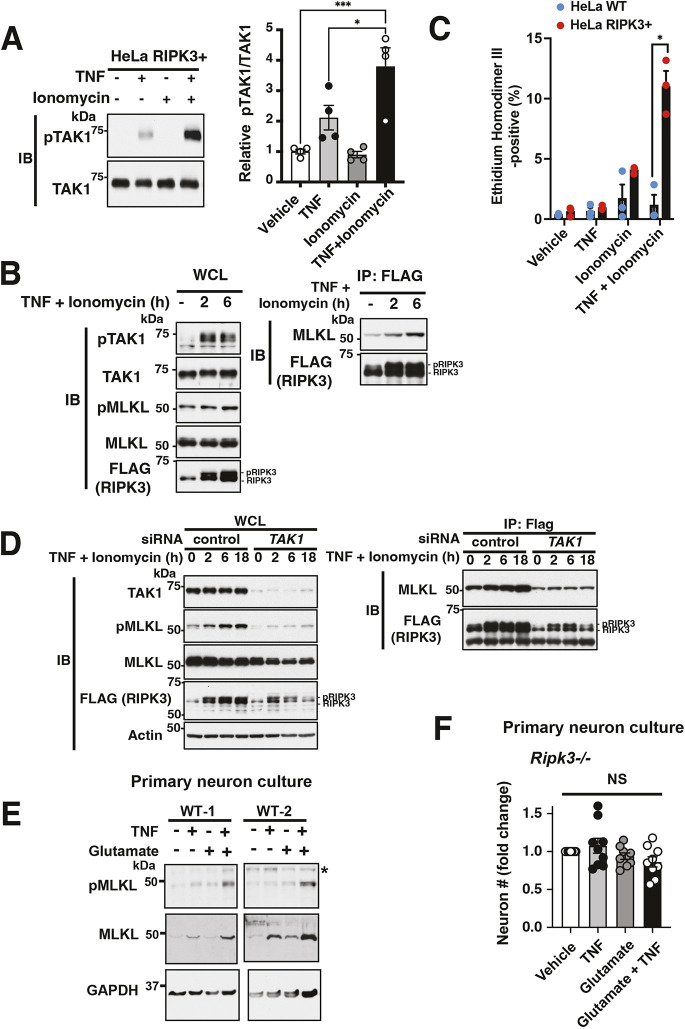
**TAK1 that is aberrantly activated by TNF and glutamate leads to necroptotic cell death.** (A) HeLa cells stably expressing FLAG–RIPK3 (HeLa RIPK3+) were treated with 10 μM ionomycin and 200 ng/ml TNF as indicated for 5 min. The activation status of TAK1 was determined by measuring the phosphorylation of TAK1 Thr-187 (pTAK1). Relative pTAK1/TAK1 levels were quantified (right graph). All data points and mean±s.e.m. are shown. (B) HeLa cells stably expressing FLAG–RIPK3 were treated with 10 μM ionomycin and 200 ng/ml TNF for the indicated time period. Immunoprecipitation with anti-FLAG antibody was conducted, and the whole-cell lysate (left panels, WCL) and the immunoprecipitates (tight panels, IP: FLAG) were analyzed by western blotting (IB) with the indicated antibodies. pMLKL, Thr357/Ser358 phosphorylated active form of MLKL; pRIPK3, phosphorylated active form of RIPK3. Representative data from two similar results is shown. (C) Wild-type HeLa (HeLa WT) cells and HeLa cells stably expressing FLAG–RIPK3 (HeLa RIPK3+) were treated with 10 μM ionomycin and 200 ng/ml TNF for 24 h. The cells were then stained with ethidium homodimer III (dead cell marker) and Hoechst 33342 (cell-permeant nuclear stain). The graph represents the percentage of ethidium homodimer III-positive dead cells from three independent experiments. (D) HeLa cells stably expressing FLAG–RIPK3 were transfected either with control or *Tak1* siRNA and cultured for 48 h. The cells were then treated with 10 μM ionomycin and 200 ng/ml TNF for the indicated time period. Immunoprecipitation with anti-FLAG antibody was conducted, and the whole cell lysate (upper panels, WCL) and the immunoprecipitates (lower panels, IP: FLAG) were analyzed by western blotting with the indicated antibodies. One result from three similar experiments is shown. (E) Primary cortical neuron cultures were treated with 50 ng/ml TNF and 10 μM glutamate as indicated for 3 days. Proteins were analyzed by western blotting with indicated antibodies. Two representative results from three independently isolated wild-type neuron cultures are shown. (F) Primary cortical neuron cultures from *Ripk3^−/−^* embryos were treated with the indicated chemicals (TNF, 50 ng/ml; glutamate, 20 μM; 5ZOZ, 300 nM; KN-62, 10 μM) for 3 days. The number of TUJ1-positive neurons from three fields/treatment was counted as is presented as a fold change over vehicle with mean±s.e.m. and all data points. Representative pictures are shown in Fig. S5B. *P<0.05; ***P<0.001; NS, not significant (*P*≥0.05) [one-way ANOVA with Tukey's multiple comparisons test (A,F); unpaired two-tailed Student's *t*-test (C)].

TNF stimulation on *Tab2*-deficient cells induces hyper-activation of TAK1, which leads to necroptotic cell death (Morioka et al., 2014). As this form of TAK1 hyper-activation is mediated by the protein kinase RIPK3 (Morioka et al., 2014), we asked whether co-stimulation with TNF and Ca^2+^ influx induced TAK1 hyper-activation requires RIPK3. To this end, we used the HeLa model cell line, which is deficient for RIPK3 expression (Su et al., 2016), and HeLa cells stably expressing FLAG-tagged RIPK3. A Ca^2+^ ionophore, ionomycin, was used to stimulate Ca^2+^ influx in these cells, as they do not express glutamate receptors. Although ionomycin treatment did not alter TAK1 activation kinetics in wild-type HeLa cells (Fig. S5A), ionomycin highly enhanced TNF-induced TAK1 activation in RIPK3-expressing cells (Fig. 5A). These treatments also induced phosphorylation of necroptosis mediators RIPK3 and MLKL (Rodriguez et al., 2016; Wang et al., 2014) (Fig. 5B, left panels). Furthermore, immunoprecipitation analysis revealed that this compound stimulation promotes complex formation between RIPK3 and MLKL, indicating activation of the necroptotic cell death pathway (Qiu et al., 2018) (Fig. 5B, right panels). Although ionomycin alone killed a small portion of HeLa cells regardless of RIPK3 expression, addition of TNF enhanced cell death only in RIPK3-expressing cells but not in RIPK3-deficient cells (Fig. 5C). Importantly, siRNA-mediated depletion of TAK1 abolished complex formation between RIPK3 and MLKL upon the compound treatment with ionomycin and TNF (Fig. 5D), indicating that Ca^2+^-dependent TNF-induced necroptosis is mediated by TAK1. To determine whether necroptosis is involved in neuronal loss, we examined MLKL activation in primary cortical neurons (Fig. 5E). Phosphorylated MLKL, as well as total MLKL protein levels, were elevated with compound treatment of TNF and glutamate. Furthermore, deletion of the necroptosis mediator RIPK3 largely blocked neuronal loss caused by TNF and glutamate co-treatment in primary cortical neurons (Fig. 5F; Fig. S5B). These results demonstrate that co-stimulation of TNF and Ca^2+^ influx elicits necroptosis through TAK1 hyper-activation leading to neuronal loss.

**Fig. 6.**
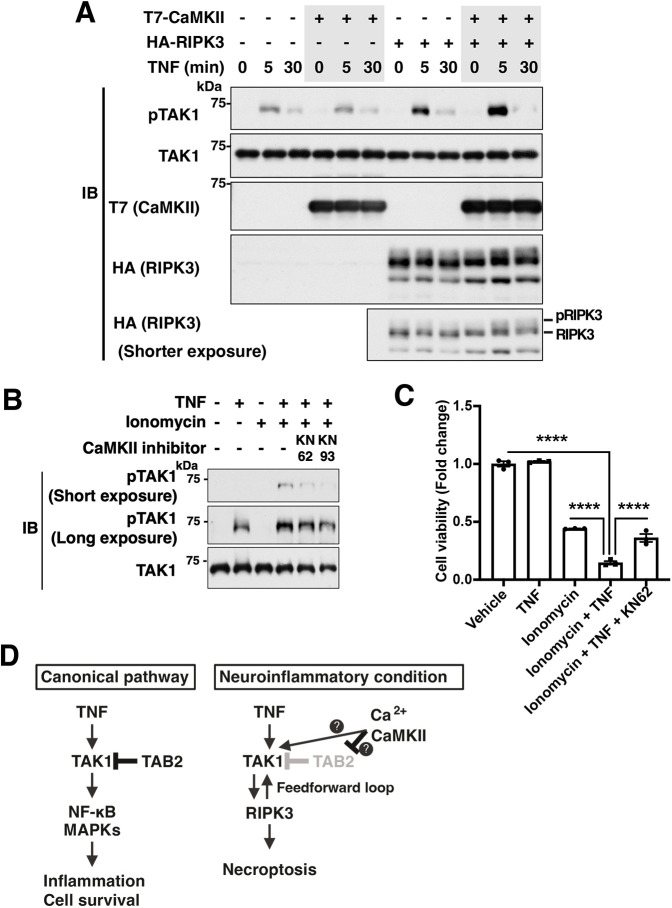
**TNF- and glutamate-induced aberrant activation of TAK1 and subsequent necroptotic cell death are mediated by CaMKII.** (A) HEK293 cells were transfected with an empty vector, T7-CaMKIIβ T286D plasmid or HA–RIPK3 plasmid as indicated and cultured for 48 h. The cells were then treated with 200 ng/ml TNF for the indicated time period. The protein lysates were analyzed by western blotting (IB) with the indicated antibodies. (B) HeLa cells stably expressing FLAG–RIPK3 were treated with the indicated chemicals (TNF, 50 ng/ml; ionomycin, 10 μM; KN62, 10 μM; KN93, 10 μM) for 5 min. The protein lysates were analyzed by western blotting with the indicated antibodies. Results in A and B are representative of two repeats. (C) HeLa cells stably expressing FLAG–RIPK3 were treated with the indicated chemicals (TNF, 50 ng/ml; ionomycin, 10 μM; KN62, 10 μM; KN93, 10 μM) for 24 h. Viability of the cells was measured by Crystal Violet staining. The graph shows fold change of cell viability (relative to the vehicle-treated control) as mean±s.e.m., *n*=3. ****P<0.0001 (one-way ANOVA with Tukey's multiple comparisons test). (D) Model of neuroinflammation-associated TAK1 aberrant activation. TAK1 normally mediates regulated inflammation but not cell death. However, under neuroinflammatory conditions, TNF and CaMKII activation coordinately hyper-activate TAK1, resulting in necroptosis.

We next investigated the mechanism by which a Ca^2+^ signal enhances TNF-induced TAK1 activation. We and others have previously shown that CaMKII members, a protein kinase family whose enzymatic activity is tightly regulated by intracellular Ca^2+^ concentration, can directly activate TAK1 (Ishitani et al., 2003; Liu et al., 2008). The CaMKII family consists of multiple isoforms that are encoded by four separate genes, *CAMKIIA*, *CAMKIIB*, *CAMKIIG* and *CAMKIID* in humans. All isoforms are structurally and functionally similar, but their expression levels vary depending on tissues (Bayer and Schulman, 2019). CaMKIIα and CaMKIIβ are highly expressed in the hippocampal neurons, and importantly, perturbation of neuronal Ca^2+^-CaMKII signaling is closely linked to the pathogenesis of AD (Murray et al., 1994; Wang et al., 2013, 2005). This prompted us to hypothesize that aberrant Ca^2+^-CaMKII signaling modulates TAK1, which switches the canonical TNF-TAK1 pathway to the TAK1 hyper-activation pathway, leading to necroptotic cellular damage. To test this hypothesis, we overexpressed a constitutively active form of CaMKIIβ (CaMKIIβ T287D) in HEK293 cells (Wang et al., 1998). Given that HEK293 cells also lack RIPK3, we co-expressed RIPK3 to induce TAK1 hyper-activation (Morioka et al., 2014). Whereas ectopic expression of CaMKIIβ T287D alone did not alter TNF-induced TAK1 activation, co-expression of RIPK3 strongly enhanced it (Fig. 6A). Conversely, treatment with pharmacological inhibitors of CaMKII, KN-62 and KN-93 (Sumi et al., 1991; Tokumitsu et al., 1990), blocked TNF and ionomycin-induced TAK1 hyper-activation (Fig. 6B) and cell death (Fig. 6C). The CaMKII inhibitor KN-62 also reduced glutamate and TNF-induced cell death in primary neurons (Fig. 4A). These results demonstrate that CaMKII is the mediator of TAK1 hyper-activation and neuronal death. We note here that expression of CaMKIIβ T287D alone did not activate RIPK3 (Fig. 6A, bottom panel, lanes 1 and 4). This suggests that CaMKII is not capable of directory activating RIPK3, but activates RIPK3 and necroptosis through modulating TAK1.

Finally, we asked whether pharmacological inhibition of TAK1 is effective in ameliorating AD pathology. The TAK1 inhibitor 5ZOZ was effective in blocking neurotoxic factor-induced primary neuron death, and 5ZOZ treatment alone exhibited no toxicity to the neurons (Fig. 4A). Furthermore, it has been reported that *Tak1* deletion in microglia results in alleviation of autoimmune inflammation caused by experimental autoimmune encephalomyelitis and post-ischemic neuroinflammation (Goldmann et al., 2013; Lu et al., 2017; Zeyen et al., 2020). These results imply that TAK1 inhibition could be further effective in ameliorating neurodegeneration as it can block both neuron death and microglial inflammation. Indeed, the TAK1 inhibitor 5ZOZ was highly effective in blocking expression of inflammatory cytokines, IL-6, IL-1β and TNF (Fig. S6). Another TAK1 inhibitor Takinib (Totzke et al., 2017) was less effective in the blockade of IL-6 and TNF, but still highly effective in blocking IL-1β production (Fig. S6). These results warrant future study of pharmacological inhibition of TAK1 and its potential to improve AD pathology.

## DISCUSSION

Necroptosis was originally identified as an alternative programed cell death pathway that is activated only by a particular combination of inflammatory ligands with apoptotic inhibitors in cultured cells, such as TNF together with pharmacological inhibitors of cellular inhibitors for apoptosis proteins (cIAPs) and caspases (Linkermann and Green, 2014). However, recent studies have demonstrated that this cell death pathway has evolved to combat against pathogen invasion (Downey et al., 2017; Sai et al., 2019; Thapa et al., 2016). Although the cell death-mediated defensive mechanism is beneficial for the host organism to prevent pathogen invasion, it can be aberrantly activated under some circumstances, which leads to undesired cell death and inflammatory tissue damage. Accumulating evidence has shown that necroptotic cell death is associated with neurodegenerative diseases, such as amyotrophic lateral sclerosis and multiple sclerosis (Degterev et al., 2019; Ito et al., 2016; Ofengeim et al., 2015; Picon et al., 2021; Re et al., 2014; Zelic et al., 2021). Our results in a cell culture system demonstrate that TAK1 mediates neuronal death in response to Ca^2+^ influx together with TNF, which is likely to be through necroptosis. However, our results in AD mouse models do not rule out the possibility that *Tak1* gene deletion in astrocytes or oligodendrocytes contributes to blockade of neuronal death. Further unidentified mechanisms other than necroptosis might also be involved, as *Ripk3* deletion did not completely block neuronal loss. Nevertheless, neuronal TAK1 is likely to be the mediator of AD-associated neuronal loss, which mainly occurs through necroptosis. These findings might provide a molecular basis for the pathogenesis of AD, which fills a current knowledge gap.

The question arises as to why Ca^2+^ signaling is linked to the necroptotic cell death pathway. Ca^2+^ plays an important role as a secondary messenger in the cytoplasm and is involved in many intracellular signaling pathways to maintain cellular homeostasis. Thus, Ca^2+^ concentrations are normally tightly regulated. However, intracellular pathogen invasion often causes elevation of intracellular Ca^2+^ concentration, which is mediated by rupture of pathogen-containing vacuoles during their invasion into the cytoplasm (Desvignes et al., 2015; Lebreton et al., 2015). The Ca^2+^-dependent cell death pathway might have evolved to combat cytoplasmic pathogens.

Blockade of TAK1 activity in many cell types induces cell death (Mihaly et al., 2014). However, as we and others have shown, *Tak1* deletion or inhibition does not trigger cell death in neurons, but rather protects them from cellular damage induced by neurotoxic factors (Goldmann et al., 2013; Sai et al., 2016). Why does TAK1 inhibition result in different outcomes depending on cell types? It has recently been reported that TAK1-inhibition-induced cell death is mediated by RIPK1 activation, which triggers downstream programmed cell death pathways, including apoptosis and pyroptosis (Geng et al., 2017; Jaco et al., 2017). This cell death-inducing mechanism is considered to be a host defense strategy against pathogens, which are capable of inhibiting components of inflammatory pathways, such as TAK1, MAP kinases and NF-κB (López-Pérez et al., 2021; Orning et al., 2018; Paquette et al., 2012). Although cell death-mediated removal of infected cells is an efficient strategy to fight against pathogens, non-proliferating cells including neurons, cannot be replaced once they are dead, and their loss is detrimental to the host. It is therefore reasonable that inhibition of inflammatory pathways by pathogens, including inhibition of TAK1, might not lead to activation of RIPK1 and subsequent initiation of cell death pathways. Nevertheless, the fact that deletion of neuronal *Tak1* blocked neuronal loss and cognitive decline without inducing cell death implies that TAK1 signaling is a potential therapeutic target in AD.

We report here that compound stimulation with TNF and glutamate (triggering Ca^2+^ influx) effectively activates TAK1 in primary neurons, whereas TNF or glutamate alone marginally activates it. TNF is an activator of TAK1; however, TNF-induced TAK1 activation is normally transient and leads to NF-κB and MAPK activation (canonical pathway) (Fig. 6D). We previously reported that TAK1 is hyper-activated when a TAK1-binding protein, TAB2, is deleted (Morioka et al., 2014). This TAK1 hyper-activation switches the pathway downstream TAK1 from the canonical pathway to a RIPK3–TAK1 activation feedforward loop leading to necroptosis (Morioka et al., 2014). In our separate study, we found that CaMKII activates TAK1 when CaMKII is overexpressed (Ishitani et al., 2003). Taken these together with our current study, we propose that CaMKII modulates TAB2 resulting in TAK1–RIPK3 necroptosis upon TNF stimulation (Fig. 6D). Further studies will be needed to define how Ca^2+^ influx switches TNF-induced TAK1 activation from the canonical inflammation pathway to the necroptosis pathway in neurons.

## MATERIALS AND METHODS

### Mice and cell culture

*Tak1*-floxed mice were described previously (Sato et al., 2005). *Nestin-Cre* transgenic mice (Zimmerman et al., 1994), *CaMKIIα-Cre* transgenic mice (Dragatsis and Zeitlin, 2000), APP/PS1 transgenic mice [strain: B6.Cg-Tg (APPswe,PSEN1dE9)85Dbo/Mmjax; Radde et al., 2006) and PS19 transgenic mice [strain: M,B6;C3-Tg (Prnp-MAPT*P301S)PS19Vle/J; Yoshiyama et al., 2007] were obtained from the Jackson Laboratories. *Ripk3^−/−^* mice (Newton et al., 2004) were used for primary cortical neuron culture. Littermate and age-matched no-Cre mice were used as control. FLAG-tagged human HeLa cells stably expressing RIPK3 were described previously (Sai et al., 2019). HeLa and HEK293 cells (ATCC) were cultured in Dulbecco's modified Eagle's medium (Genesee Scientific) supplemented with 10% bovine growth serum (Hyclone) and 50 IU/ml penicillin-streptomycin (Genesee Scientific) at 37°C in 5% CO_2_. All animal experiments were conducted with the approval of the North Carolina State University Institutional Animal Care and Use Committee.

### Antibodies, plasmids and reagents

Anti-TAK1 antibody was described previously (Ninomiya-Tsuji et al., 1999). Anti-phospho TAK1 (Thr187) (Cell Signaling Technology, 1:1000), anti-β-actin (Sigma-Aldrich, AC15, 1:10,000), anti-MLKL (abcam, EPR17514 and 3H1, 1:1000), anti-phospho-human MLKL (Thr357/Ser358, 1:1000; abcam, EPR9514), anti-human Tau (Santa Cruz Biotechnology, Tau-13, 1:100), anti-phospho-human Tau (Ser202, Thr205) (Invitrogen, AT8, 1:50), anti-NeuN (Sigma-Aldrich, MAB377, 1:100), anti-TUJ1 (BioLegend, TUBB3, 1:1000), anti-GFAP (abcam, ab7260, 1:1000), anti-Iba1 (Wako, 019-19741, 1:500), anti-FLAG (Sigma-Aldrich, M2, 1:1000), anti-T7 (Sigma-Aldrich, 69522, 1:1000) and anti-HA (Covance, HA.11, 1:1000) antibodies were used. HA-tagged human RIPK3 and C-terminally FLAG-tagged human MLKL plasmids were kind gifts from Dr Xiaodong Wang (Department of Biochemistry, University of Texas Southwestern Medical Center, USA; He et al., 2009). Rat CaMKIIβ T287D was subcloned into pCMV-T7 vector (Takaesu et al., 2000) to generate pCMV-T7-CaMKIIβ. Plasmids were transfected using TransIT-X2 Reagent (Mirus Bio LLC). DAPI (MilliporeSigma), TNF (PeproTech), ionomycin (calcium salt) (MilliporeSigma), ethidium homodimer III (Biotium), Hoechst 33342 (Thermo Fisher Scientific), L-Glutamic acid (Glutamate) monosodium salt (SAFC), KN-62 (MilliporeSigma) and KN-93 (MilliporeSigma) were used. TAK1 protein kinase inhibitors 5ZOZ and Takinib were also used (Ninomiya-Tsuji et al., 2003; Totzke et al., 2017).

### Western blotting and immunoprecipitation

Protein extracts from cultured cells and the mouse brain tissues were prepared using an extraction buffer containing 20 mM HEPES pH 7.4, 150 mM NaCl, 12.5 mM β-glycerophosphate, 1.5 mM MgCl_2_, 2 mM EGTA, 10 mM NaF, 2 mM DTT, 1 mM Na_3_VO_4_, 1 mM phenylmethylsulfonyl fluoride, 20 μM aprotinin and 0.5% Triton X-100. For immunoprecipitation, cell lysates were prepared in the extraction buffer described above. FLAG-tagged RIPK3 proteins were immunoprecipitated with anti-FLAG antibodies (1:1000) with Protein G Sepharose 4 fast flow (GE Healthcare) for 2 h at 4°C. The resulting immune complexes were washed three times with PBS. The extracts and immunoprecipitates were resolved on SDS-PAGE gels and transferred onto Hypond-P membranes (GE Healthcare). The membranes were immunoblotted with the indicated antibodies, and the bound antibodies were visualized with horseradish peroxidase-conjugated antibodies against rabbit or mouse IgG using the Pierce SuperSignal™ West ECL western blotting systems (Thermo Fisher Scientific). ImageJ software was used for quantifications of the band intensity. Original western botting images used in the figures are shown in the supplementary blot transparency figure (Fig. S7).

### Immunofluorescence microscopy analysis

For immunofluorescence staining of the mice brain tissues, the isolated tissues were fixed with 4% paraformaldehyde for 24 h, then stored in 70% ethanol at 4°C. The fixed tissues were embedded in paraffin. Paraffin sections (5 μm) were blocked with PBS containing 3% bovine serum albumin (Santa Cruz Biotechnology) for 30 min at room temperature and incubated with primary antibodies followed by incubation with either anti-rabbit or mouse IgG conjugated to Alexa Fluor 488 or 594 (Thermo Fisher Scientific). For immunofluorescence staining of cultured cells, the cells were fixed with 10% formalin in PBS for 10 min, blocked with PBS containing 3% bovine serum albumin for 30 min at room temperature, then incubated with the primary antibodies followed by incubation with either anti-rabbit or mouse IgG conjugated with Alexa Fluor 488 or 594. The samples were examined by a fluorescence microscope (model BX41; Olympus) and camera (model DP80; Olympus) at room temperature.

### Preparation of the mouse brain tissues

Mice were euthanized by means of CO_2_ inhalation followed by cervical dislocation. Left brain hemispheres were fixed with 4% paraformaldehyde for 24 h, then stored in 70% ethanol at 4°C for immunohistochemistry. The hippocampus, the prefrontal cortex and the cerebellum of the right brain hemispheres were carefully dissected out. For preparation of protein extracts, the isolated brain tissues were homogenized using glass dounce homogenizers in an extraction buffer containing 20 mM HEPES pH 7.4, 150 mM NaCl, 12.5 mM β-glycerophosphate, 1.5 mM MgCl_2_, 2 mM EGTA, 10 mM NaF, 2 mM DTT, 1 mM Na_3_VO_4_, 1 mM phenylmethylsulfonyl fluoride, 20 μM aprotinin and 0.5% Triton X-100. An RNeasy kit (Qiagen) was used for preparation of RNA extracts.

### Measuring thickness of neuron layers in the dentate gyrus

Paraffin sections of the hippocampus were prepared from 9- to 12-month-old mice of the indicated genotypes. Immunohistochemistry using NeuN antibody was conducted on the paraffin sections of hippocampus (one section/mouse) to visualize neuron layers. To calculate thickness of dorsal dentate gyrus (DG) neuron layer, two areas (11,561 μm^2^ each) of a segment of the DG adjacent to the crest were selected from each wing and measured using ImageJ software. The NeuN-positive areas were then divided by its base length to calculate average thickness of the neuron layers.

### Primary neuron culture

Primary neurons were isolated from the cortex of embryonic day 14 to 16 mice. Briefly, the isolated cortices (meninges removed) were treated with 3 mg/ml papain (Worthington Biochemical) for 10 min in 37°C water bath, triturated by pipetting up to 10 times, and plated onto poly-D-lysine (Gibco)-coated 8-well chambers (iBidi) at a concentration of 10^5^ cells per well. The neurons were cultured in Neurobasal medium (Gibco) supplemented with 0.25× Glutamax (Gibco), 1× B27 supplement (Gibco Thermo Fisher Scientific), 10% bovine growth serum (HyClone GE Healthcare Life Sciences) and 50 IU/ml penicillin-streptomycin for 24 h. The medium was then replaced with Neurobasal medium supplemented with 0.25× Glutamax, 1× B27 and 50 IU/ml penicillin-streptomycin (no bovine growth serum). The neurons were cultured for at least 5 days *in vitro* prior to the treatment and analysis.

### Counting primary neurons, astrocytes and microglia

Primary neurons were treated with the indicated chemicals for 3 days, then fixed with 10% formalin in PBS. The fixed neurons were blocked with PBS containing 3% bovine serum albumin (Santa Cruz Biotechnology) for 30 min at room temperature, and then incubated with anti-TUJ1 antibody followed by incubation with anti-mouse IgG conjugated to Alexa Fluor 594, and DAPI. Neuron number was determined by counting TUJ1-positive cells from three randomly photographed areas for each sample. For counting glia cells in the DG of hippocampus, paraffin sections of the hippocampus were prepared from 9- to 12-month-old mice of the indicated genotypes. Immunohistochemistry using anti-GFAP antibody and -Iba1 antibody was conducted on the paraffine sections of hippocampus to visualize astrocytes and microglia respectively. Glia cell number was determined by counting GFAP (Astrocytes) or Iba1 (Microglia) positive cells from an area (80,000 μm^2^) of the DG for each mouse.

### Morris water maze

The test was conducted in a circular white pool (114 cm diameter) with opacified water (30 cm deep) at 25°C. A hidden platform (13 cm diameter, 1 cm below the water surface) was placed in the pool. The mice were placed in the pool and allowed to swim freely for 60 s to search for the platform. If the mice failed to find the platform in 60 s, they were manually placed onto the platform. The mice were then allowed to stay on the platform for 15 s, dried and rested in their originally housed cage at least 10 min before the next trial. The mice were trained with four trials per day, which were started from four cardinal points of the compass for 6 consecutive days. All the swimming paths and the times taken for reaching the platform (escape latency) were recorded. At 24 h after the last training session (day 7), the platform was removed, and the mice were allowed to swim for 60s. During this test session, all mouse movements were recorded by a video camera placed above the pool. For quantification of platform crossing, the number of times the mice crossed the area where the platform had been located (13 cm diameter) during the test session was counted using AnimalTracker (Gulyás et al., 2016). For quantification of the percentage of time spent in the platform area, time spent in the area where the platform had been located (30% of the whole area, 62 cm diameter) during the test session was compared with averaged time spent in the rest of the areas.

### Novel object recognition test

The test was conducted in an open opaque cage (45 cm×23 cm). The first day of the experiment (habituation session), the mice were placed in the empty cage, facing the wall, and allowed to freely explore the cage for 5 min. The mice were then returned to their original housing cage. The second day of the experiment (familiarization session), the mice were placed in the cage containing two identical objects, placed 5 cm away from the walls. The mice were allowed to freely explore the cage until total contact time with the objects (sniff or touch) reached 20 s, then returned to their original housing cage. If the mice did not contact to the objects for 20 s during 10 min period, they were returned to their original housing cage. The third day of the experiment (test session), the mice were placed in the cage containing one familiarized object and one novel object, placed 5 cm away from the walls. The mice were allowed to explore freely in the cage until total contact time with the objects (sniff or touch) reached to 20 s, then returned to their original housing cage. If the mice did not contact with the objects for 20 s during 10 min period, they were returned to its original housing cage. During the test session, all mouse movements were recorded by a video camera placed above the cage. Contact time with familiar object (T_F_) and novel object (T_N_) were measured, and Discrimination Index (DI) was calculated by the following formula: [DI=(T_N_−T_F_)/(T_N_+T_F_)]. The cage was wiped with 70% isopropanol between each experimental step.

### Crystal Violet staining and cell death assay

Cells were seeded on 24-well plates at a concentration of 6.25×10^4^ cells per well. The cells were treated with the indicated chemicals for 24 h. Floating dead cells were washed out with PBS and adherent cells were fixed with 10% formalin in PBS. For crystal violet assay, the cells were stained with 0.1% Crystal Violet. The dye was eluted in 50% ethanol, 0.1 M sodium citrate and analyzed at 595 nm using a SmartSpec™ 3000 machine (Bio-Rad). For the cell death assay, the cells were stained with ethidium homodimer III and Hoechst 33342. For quantification of dead cells, randomly photographed pictures for each sample were used. At least 800 cells were analyzed for each sample, and total number of the cells (Hoechst-positive) and number of dead cells (ethidium homodimer III-positive) were quantified using ImageJ software. To calculate ratio of dead cells, the number of dead cells was divided by the total cell number.

### Knockdown of *Tak1*

Custom siRNAs targeting *Tak1* and control siRNA were generated by Sigma (TAK1, 5′-GGCAAAGCAACAGAGUGAAUCUGGA-3′; control siRNA, 5′-UUCUCCGAACGUGUCACGU-3′). HeLa cells were transfected with the siRNAs using TransIT-X2 Reagent (Mirus Bio LLC).

### Primary microglia culture

Primary microglia were isolated from the cortex of postnatal day 2 to 4 mice. Briefly, the isolated cortices (meninges removed) were treated with papain for 20 min in 37°C water bath, triturated by pipetting up to 15 times, and 1×10^7^ cells were plated onto poly-D-lysine-coated T75 flasks (Mixed glial culture). The mixed glial cells were cultured in Dulbecco's modified Eagle's medium supplemented with 10% bovine growth serum and 50 IU/ml penicillin-streptomycin. After 7 days, the cultures were shaken vigorously (180 rpm on a rotary shaker for 1 h at 37°C) to isolate microglia. The isolated microglia were collected and plated onto 24-well plates at a concentration of 10^5^ cells per well and cultured in Dulbecco's modified Eagle's medium supplemented with 10% bovine growth serum, 50 IU/ml penicillin-streptomycin and 30% conditioned medium from L929 cells (ATCC). The microglia were cultured for 3 days *in vitro* before treatment and analysis.

### Quantitative real-time PCR analysis

Total RNA was isolated from the mouse brain tissues and primary microglia using an RNeasy kit (Qiagen) and transcribed into cDNA using MultiScribe reverse transcriptase (Thermo Fisher Scientific). Expression levels of TNF, IL-6, IL-1β, CCL2 and CCL3-encoding mRNAs were determined by quantitative real-time PCR (iTaq Universal SYBR Green System, Bio-Rad) and normalized to the level of GAPDH. The following primers were used: mouse TNF-forward, 5′-GTCCCCAAAGGGATGAGAAGTT-3′; mouse TNF-reverse, 5′-CTCCTCCACTTGGTGGTTTG-3′; mouse IL-6-forward, 5′-TCCGGAGAGGAGACTTCACA-3′; mouse IL-6-reverse, 5′-TGCCATTGCACAACTCTTTTC-3′; mouse IL-1β-forward, 5′-TGCCACCTTTTGACAGTGATG-3′; mouse IL-1β-reverse, 5′-TGATGTGCTGCTGCGAGATT-3′; mouse CCL2-forward, 5′-CACTCACCTGCTGCTACTCA-3′; mouse CCL2-reverse, 5′-GCTTGGTGACAAAAACTACAGC-3′; mouse CCL3-forward, 5′-TCCCAGCCAGGTGTCATTTTC-3′; mouse CCL3-reverse, 5′-TCAGGCATTCAGTTCCAGGTC-3′; mouse GAPDH-forward, 5′-GAAGGTCGCTGTGAACGGA-3′; mouse GAPDH*-*reverse, 5′-GTTAGTGGGGTCTCGCTCCT-3′.

### Statistical analyses

The box and whisker graphs represent the median (middle line), the 25th and 75th percentiles (box), and the minimum and maximum date points (whiskers), and all data points are shown. Statistical analyses were performed using the one-way ANOVA with Tukey's multiple comparisons test, or the unpaired two-tailed Student's *t*-tests with equal distributions. Significance is denoted as *P<0.05; **P<0.01; ***P<0.001; ****P<0.0001; NS, not significant when *P*>0.05.

## Supplementary Material

10.1242/joces.260102_sup1 Supplementary information
