# Supplementary material for: Aberrantly activated TAK1 links neuroinflammation and neuronal loss in Alzheimer's disease mouse models
Source: J Cell Sci. 2023 Mar 13;136(6):jcs260102. doi: 10.1242/jcs.260102 (PMC10112982; doi:10.1242/jcs.260102)
Supplement: Supplementary information [file joces-136-260102-s1.pdf]

Fig. S1

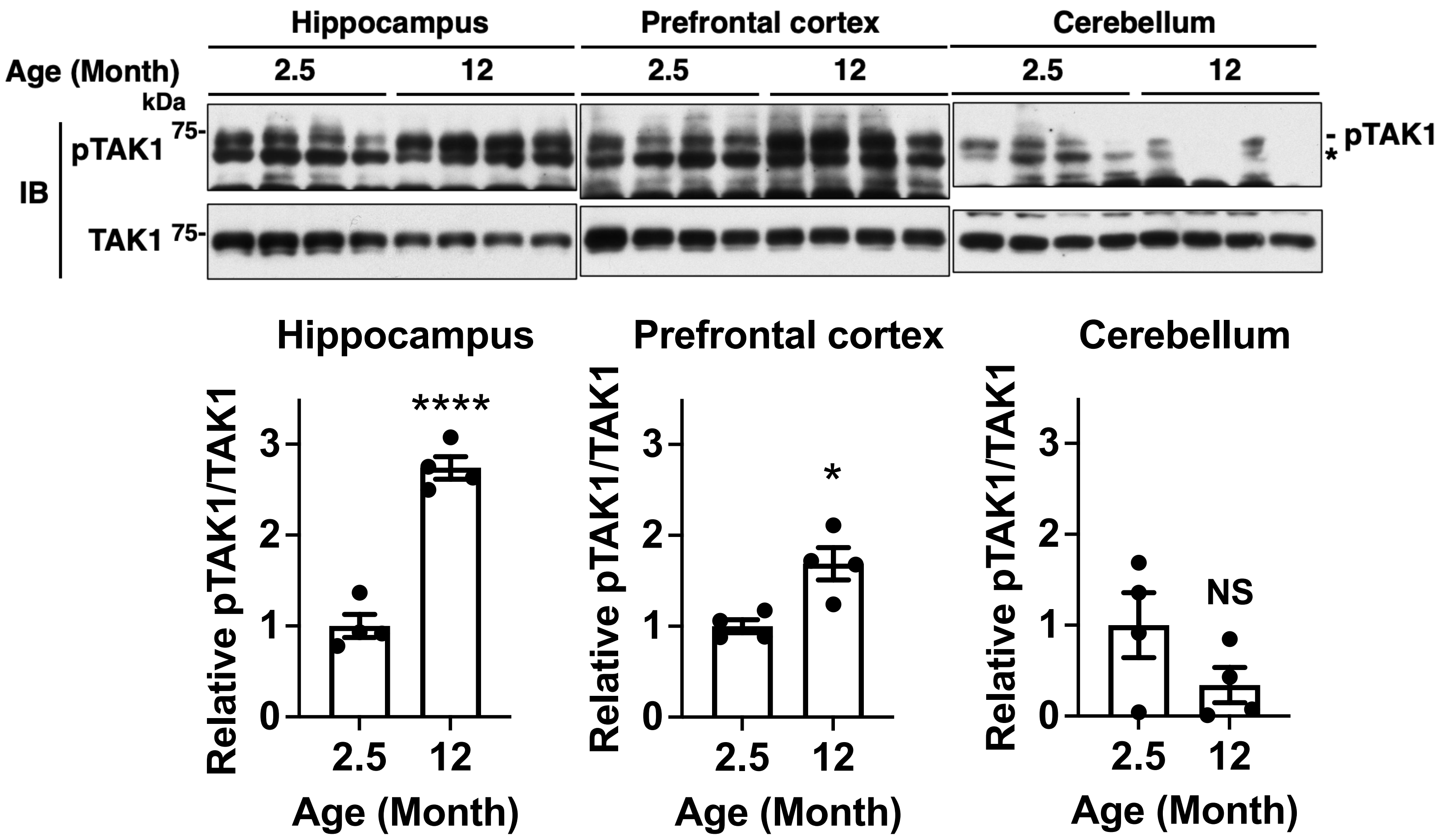

Fig. S1. TAK1 activity is elevated in the hippocampus and prefrontal cortex of aged mice Top panels: Protein extracts of the indicated brain area from 2.5- and 12-month-old wild type mice were analyzed by Western blotting with the indicated antibodies. Each lane represents an individual animal. Activation status of TAK1 was determined by phosphorylation of TAK1 Thr-187 (pTAK1). *, non-specific band. Bottom graphs: Relative pTAK1/TAK1 levels were quantified. All data points are shown. mean ± SEM; *, $p < 0.05$; ****, $p < 0.0001$; NS, not significant when $p \geqq 0.05$.

Fig. S2

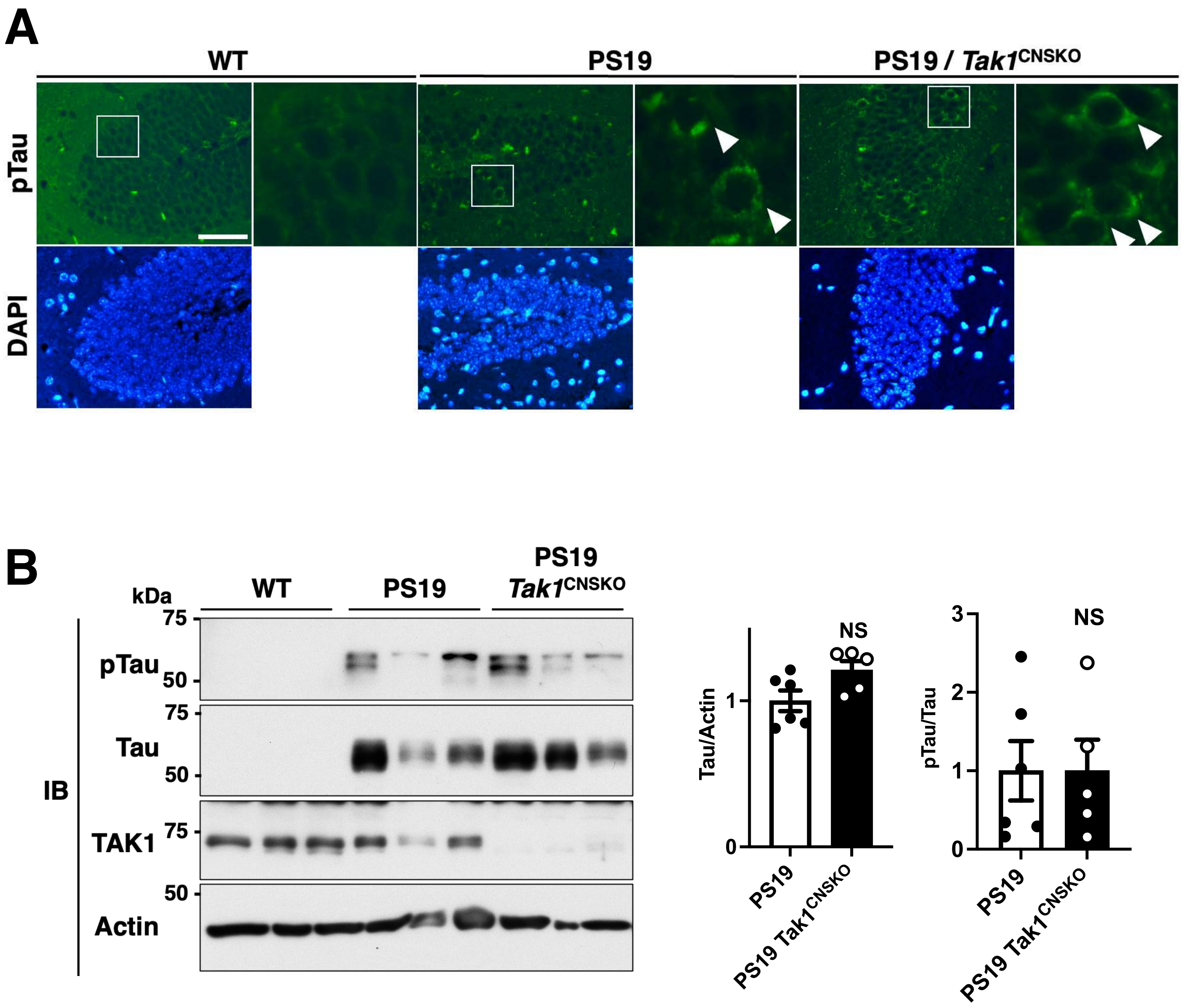

Fig. S2. Accumulation of phospho-Tau is not altered by CNS-specific *Tak1* deficiency

(A) Representative pictures of immunohistochemistry with anti-phospho-human-Tau (Ser202, Thr205) antibody conducted on hippocampal DG sections from 8- to12-month-old wild type (WT) and PS19 mice with or without CNS-specific *Tak1* deficiency (*Tak1$^{flox/flox}$ Nestin-Cre, Tak1^{CNSKO}*). Arrowheads indicate accumulation of pTau in the neurons. Scale bars, 50 µm. (B) Left panels: Representative Western blotting with the indicated antibodies of hippocampal protein extracts from 8- to 12-month-old wild type (WT) and CNS-specific *Tak1* deficient (*Tak1$^{flox/flox}$ Nestin-Cre, Tak1^{CNSKO}*) mice. Each lane represents an individual animal. Right graphs: Relative Tau/Actin and pTau/Tau levels were quantified using the ImageJ software. All data points are shown. mean ± SEM; NS, not significant when p ≧ 0.05.

Journal of Cell Science • Supplementary information

Fig. S3

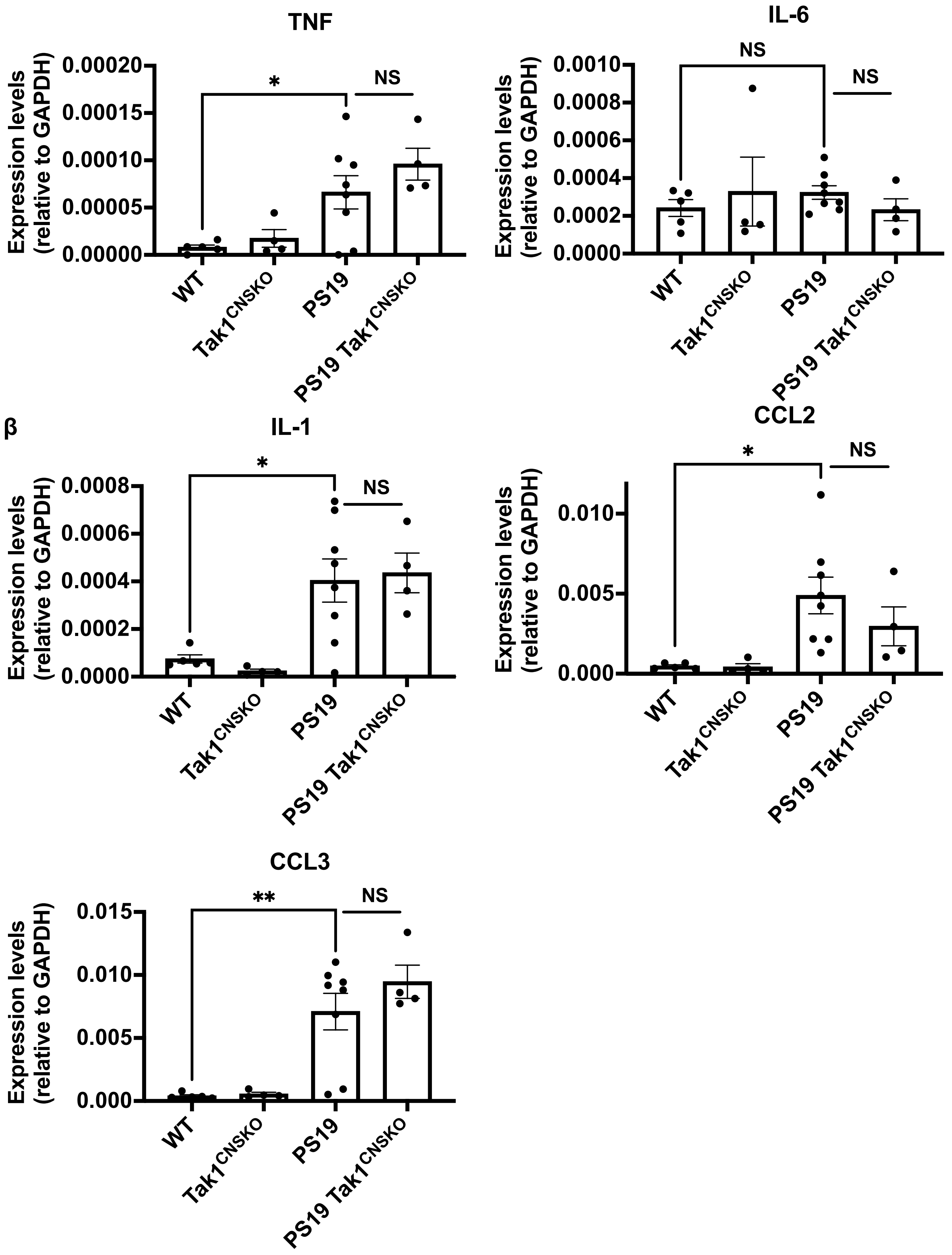

Fig. S3. AD-associated neuroinflammation is not altered by CNS-specific Tak1 deficiency

Expression levels of inflammatory cytokines and chemokines (relative to GAPDH) in the hippocampus from 8- to 12-month-old wildtype (WT) PS19 mice with or without CNS-specific *Tak1* deficiency ($\mathit{Tak1}^{flox/flox}$ *Nestin-Cre,* $\mathit{Tak1}^{CNSKO}$) were analyzed by quantitative real-time PCR. All data points are shown. WT, n = 5; $Tak1^{CNSKO}$, n = 4; PS19, n = 8; PS19 Tak1CNSKO, n = 4; mean ± SEM; *, $p < 0.05$; **, $p < 0.01$; NS, not significant when p≧0 .05.

Journal of Cell Science • Supplementary information

Fig. S4

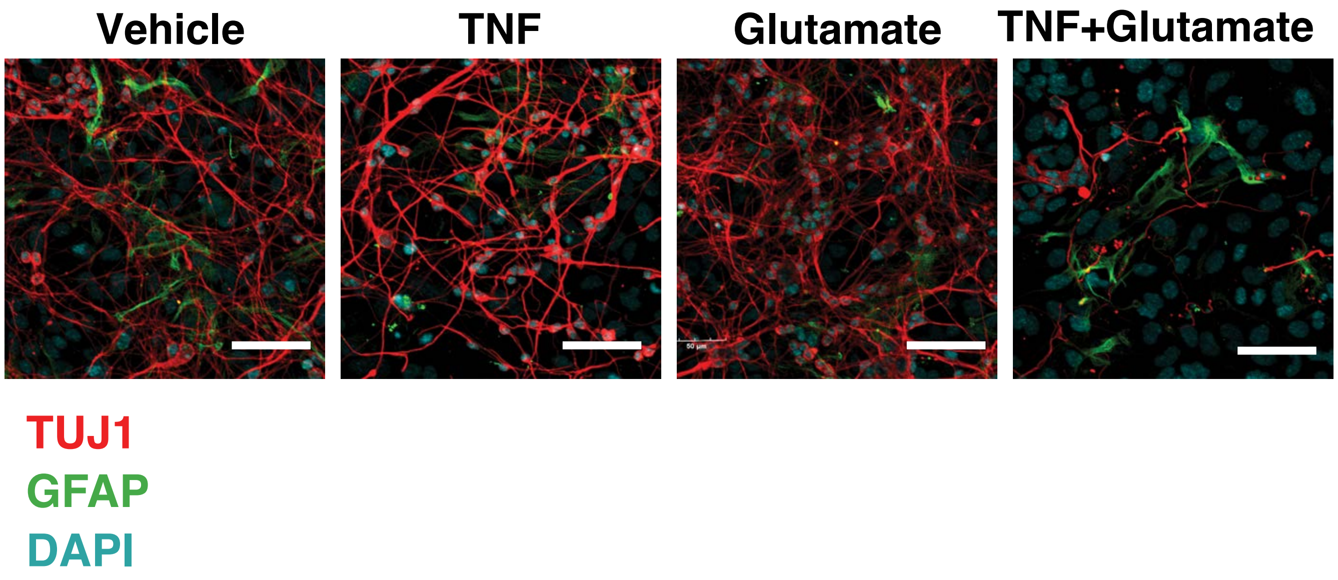

Fig. S4. Compound treatment of TNF and glutamate reduced number of neurons in primary cortical neuron cultures.

Wild type mice embryonic neurons were treated with the indicated chemicals (TNF, 50 ng/ml; Glutamate, 20 µM) for 3 days. Neurons were fixed and stained with anti-TUJ1 and anti-GFAP antibodies and DAPI. Scale bars, 50 µm.

Journal of Cell Science • Supplementary information

Fig. S5

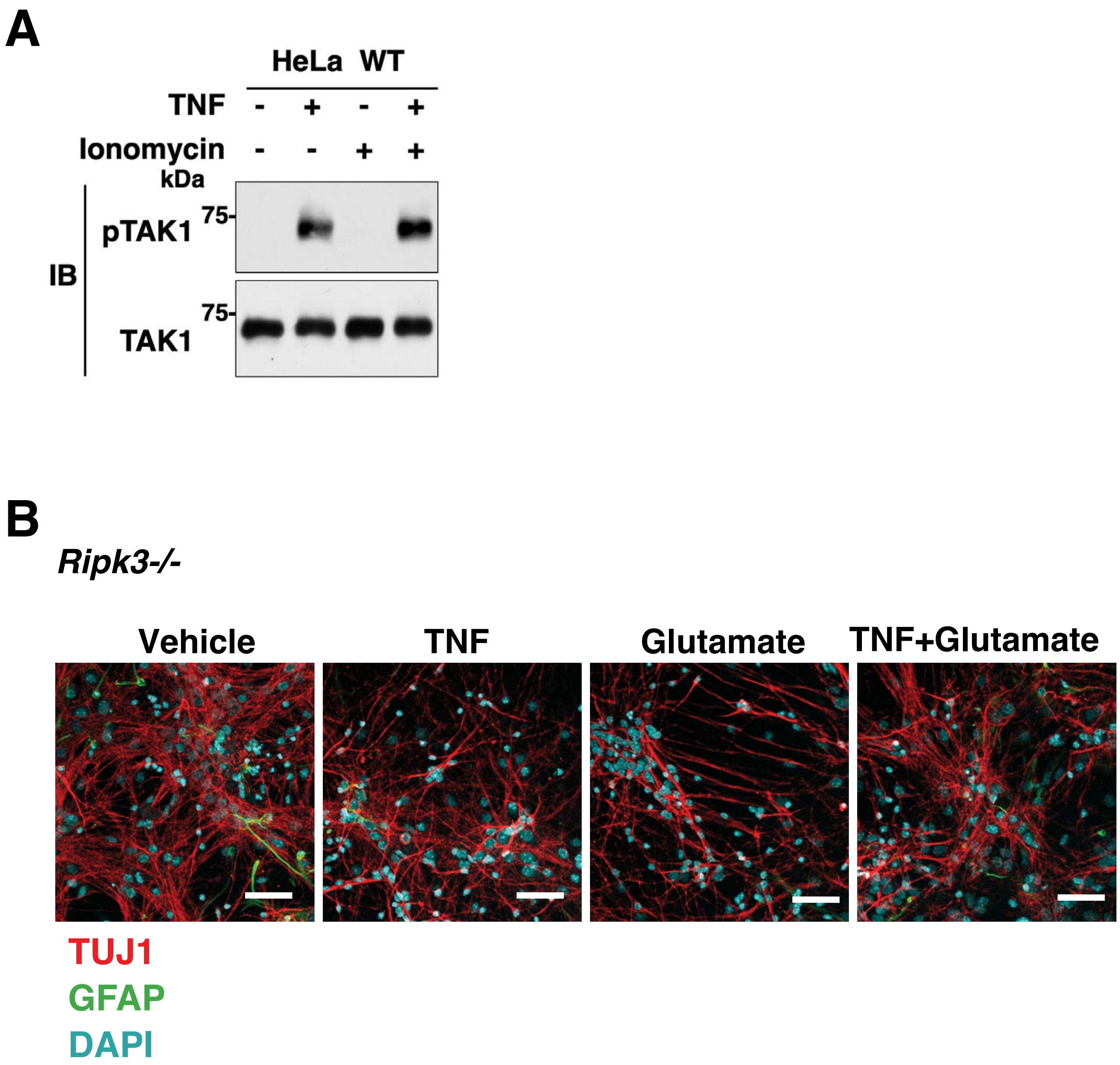

Fig. S5. (A) Compound treatment of TNF and glutamate does not cause aberrant activation of TAK1 in Ripk3-deficient HeLa cells
HeLa (HeLa WT) cells were treated with 10 µM Ionomycin and 200 ng/ml TNF as indicated for 5 min. Activation status of TAK1 was determined by phosphorylation of TAK1 Thr-187 (pTAK1).
(B) RIPK3 deletion blocks TNF + Glutamate-induced neuron loss in primary cortical cultures Ripk3-/- embryonic neuron cultures were treated with TNF, 50 ng/ml and Glutamate, 20 µM as indicated for 3 days. Neurons were fixed and stained with anti-TUJ1 and anti-GFAP antibodies and DAPI. Scale bars, 50 µm.

Journal of Cell Science • Supplementary information

Fig. S6

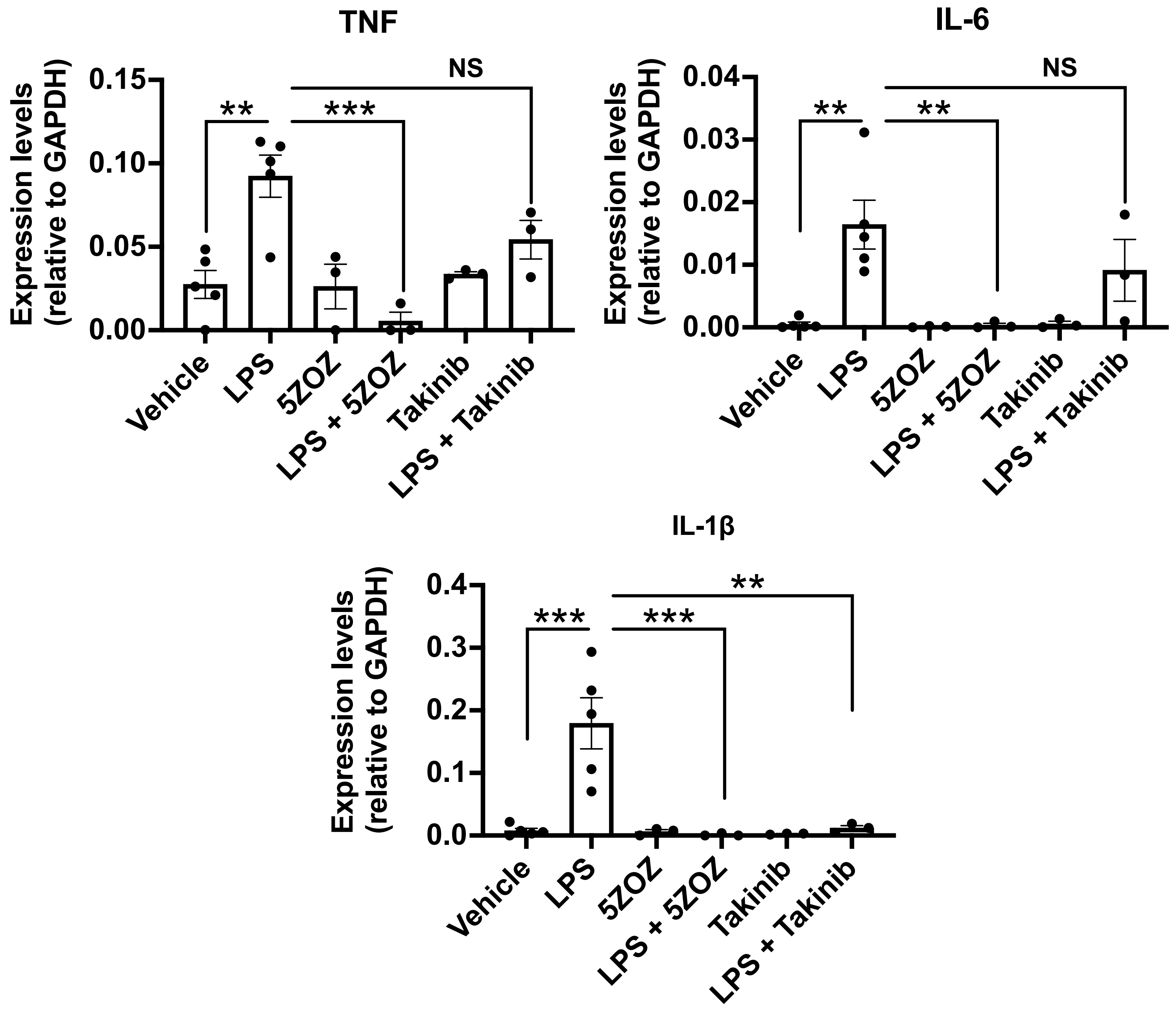

Fig. S6. Pharmacological inhibition of TAK1 ameliorates inflammation by activated microglia Expression levels of inflammatory cytokines (relative to GAPDH) in the primary cultured microglia treated with the indicated chemicals (LPS, 100 ng/mL; 5ZOZ, 300 nM; Takinib, 10 μM) for 24 h were analyzed by quantitative real-time PCR. All data points are shown. Vehicle, n = 5; LPS, n = 5; 5ZOZ, n = 3; LPS + 5ZOZ Z, n = 3; Takinib, n = 3; LPS + Takinib, n = 3; mean ± SEM; **, $p < 0.01$; ***, $p < 0.001$; NS, not significant when p≧0 .05.

Fig. S7 Blot transparency_1

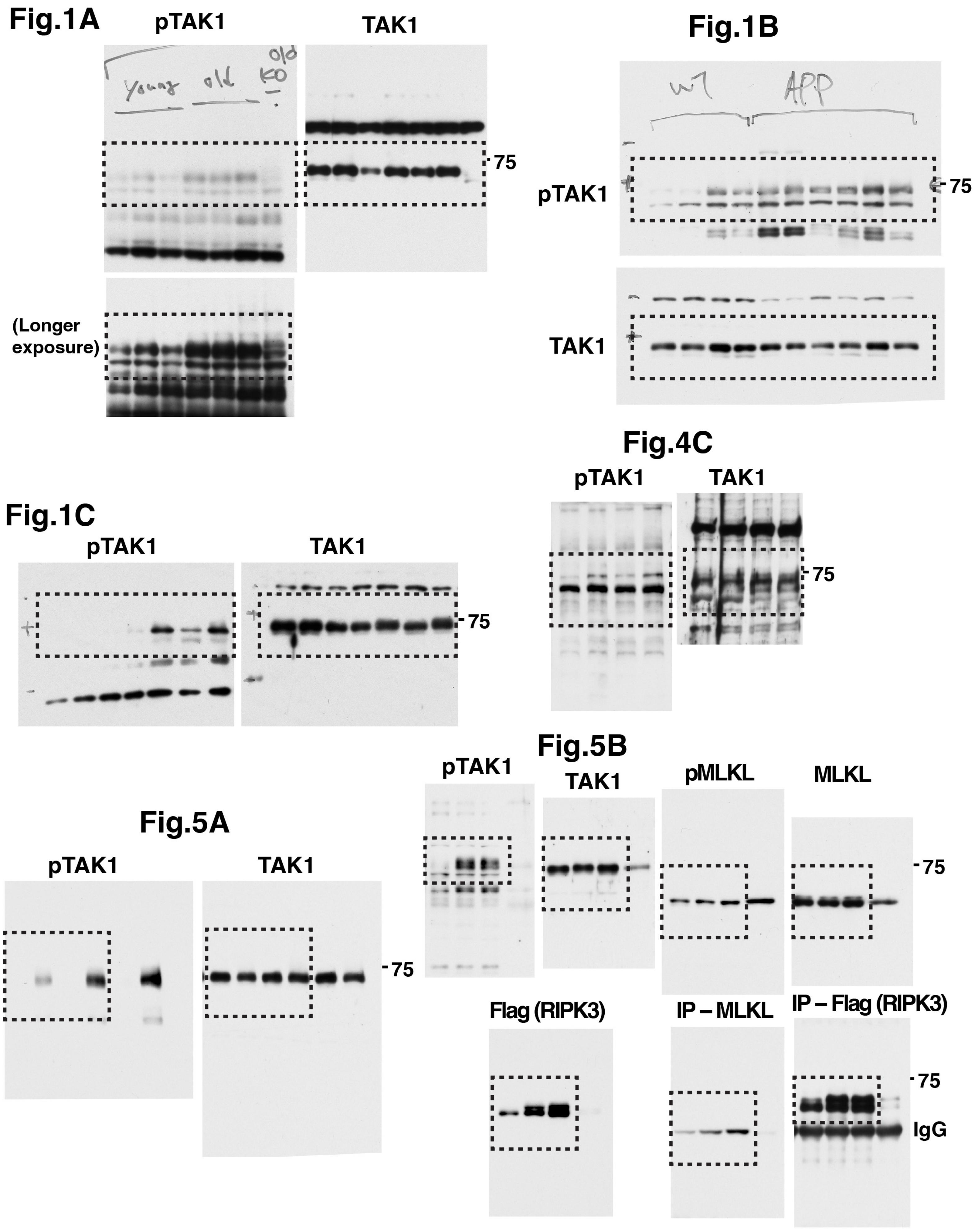

Journal of Cell Science • Supplementary information

Fig. S7 Blot transparency_2

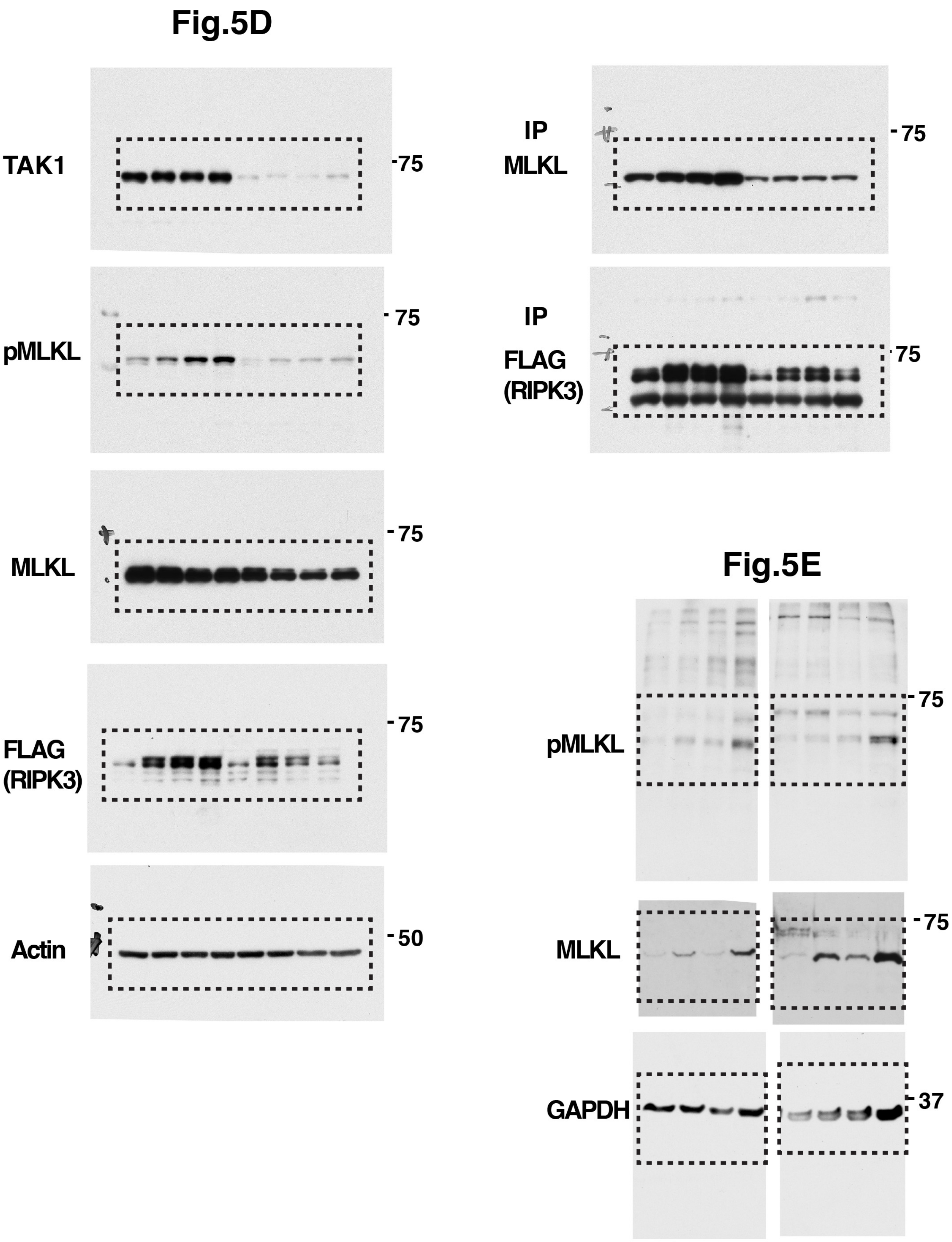

Journal of Cell Science • Supplementary information

Fig. S7 Blot transparency_3

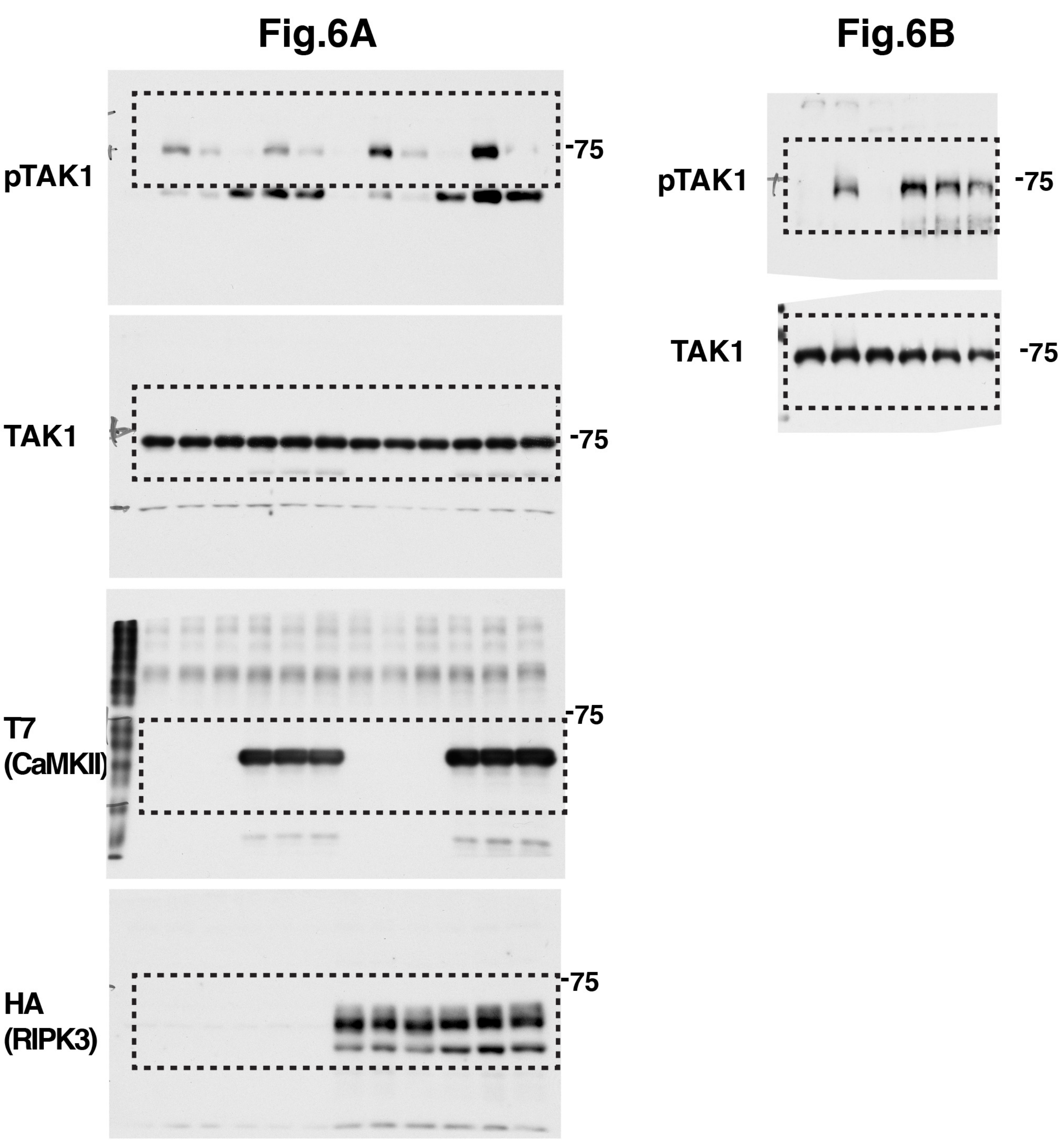

Journal of Cell Science • Supplementary information
