# Supplementary material for: Aberrantly activated TAK1 links neuroinflammation and neuronal loss in Alzheimer's disease mouse models
Source: J Cell Sci. 2023 Mar 13;136(6):jcs260102. doi: 10.1242/jcs.260102 (PMC10112982; doi:10.1242/jcs.260102)
Supplement: Supplementary file 1 [file joces-136-260102_review_history.pdf]

Journal of Cell Science

Aberrantly activated TAK1 links neuroinflammation and neuronal loss in Alzheimer's disease mouse models

Kazuhito Sai, Aoi Nakanishi, Kimberly M. Scofield, Debra A. Tokarz, Keith E. Linder, Todd J. Cohen and Jun Ninomiya-Tsuji
DOI: 10.1242/jcs.260102

Editor: Giampietro Schiavo

Review timeline

Original submission:	8 April 2022
Editorial decision:	19 May 2022
First revision received:	12 December 2022
Editorial decision:	11 January 2023
Second revision received:	23 January 2023
Accepted:	27 January 2023

Original submission

First decision letter

MS ID#: JOCES/2022/260102

MS TITLE: Aberrantly activated TAK1 links neuroinflammation and neuronal loss in Alzheimer's disease mouse models

AUTHORS: Kazuhito Sai, Kimberly M Scofield, Debra A Tokarz, Keith E Linder, Todd J Cohen, and Jun Ninomiya-Tsuji
ARTICLE TYPE: Research Article

We have now reached a decision on the above manuscript.

To see the reviewers' reports and a copy of this decision letter, please go to: https://submit-jcs.biologists.org and click on the 'Manuscripts with Decisions' queue in the Author Area. (Corresponding author only has access to reviews.)

As you will see, the reviewers raise a number of substantial criticisms that prevent me from accepting the paper at this stage. They suggest, however, that a revised version might prove acceptable, if you can address their concerns. If you think that you can deal satisfactorily with the criticisms on revision, I would be pleased to see a revised manuscript. We would then return it to the reviewers.

Please ensure that you clearly highlight all changes made in the revised manuscript. Please avoid using 'Tracked changes' in Word files as these are lost in PDF conversion.

I should be grateful if you would also provide a point-by-point response detailing how you have dealt with the points raised by the reviewers in the 'Response to Reviewers' box. Please attend to all of the reviewers' comments. If you do not agree with any of their criticisms or suggestions please explain clearly why this is so.

© 2023. Published by The Company of Biologists under the terms of the Creative Commons Attribution License (https://creativecommons.org/licenses/by/4.0/).

Reviewer 1

Advance summary and potential significance to field

In the current study, the authors found that hippocampal TAK1 is crucial to induce neuroinflammation and neuronal toxicity in Alzheimer's disease mice models, and involved in the induction of cognitive dysfunction. Furthermore, they also confirmed that co-treatment of primary cultured neurons with TNF and glutamate induced cell death through the induction of phosphorylation of TAK1. In addition these responses are regulated by CaMKII. In the current study, the experiments were well performed extensively. However, I have some concerns, and the authors should improve their manuscript to be acceptable.

Comments for the author

1. In the current study, the authors express neuronal death observed in this study as necroptosis. By contrast, they mentioned "TAK1 mediates programmed cell death pathways including apoptosis, pyroptosis and necroptosis." in the introduction section. Exactly, although they found the phosphorylation of both MLKL and RIPK3, which are crucial in the induction of necroptosis, it is somewhat of an overestimation to characterize the cell death observed in the current study as necroptosis, based on these findings alone. It is necessary to improve.

2. the author used the one-way ANOVA with Tukey's multiple comparisons test in statistic analysis. In Fig. 4, the number of data is three, but in this case of this analysis, the number of data should be more than four.

3. It is necessary to mention why the concentration of TNF used differs between Fig. 4A and 4B.

4. In Fig. 4C or 5, it is necessary to add quantitative evaluation of Western blots.

5. In Fig. 5B, the authors should examine whether treatment with either TNF or ionomycin alone induce the phosphorylation of MLKL and RIPK3.

6. In Fig. 5D, deletion of TAK1 blocked the the phosphorylation of MLKL and RIPK3. These findings led to speculate that TAK1 might mediate activities of MLKL and RIPK3. By contrast, in Fig. 6, the overexpression of RIPK3 increased the phosphorylation of TAK1. Thus, the authors should discuss these pathways about the regulation of TAK-RIPK3.

7. In addition, treatment with ionomycin alone did not induce phosphorylation of TAK1, but co-treatment with TNF and ionomycin synergistically induced. Then what mechanisms are involved in this regulation? It is necessary to examine and discuss more. I recommend to examine how the phosphorylation of CaMKII changes under the treatment conditions described above.

8. I recommend to add a figure summarized the current findings.

Reviewer 2

Advance summary and potential significance to field

The paper from Sai and colleagues describes an interesting link between the inflammation related mitogen activated protein kinase kinase kinase TAK1 and neuronal cell death, with a focus on Alzheimer's disease.

Interestingly, TAK1 is hyperactivated in aged mice and in APP/PS1 and PS19 (tau) models, and the neuron-specific depletion of TAK1 is able to revert well-known deficits present in these models such as spatial memory in APP/PS1 and neuronal loss in PS19. In separate experiments they show that TAK1 can be hyperactivated by co application of glutamate or Ionomycin and TNF in cell lines, a treatment that induces neuronal death. TAK1 activation appears to be dependent on RIPK3/MLKL and on CamKIIb.

The results are of good quality and interesting, however I have several concerns.

© 2023. Published by The Company of Biologists under the terms of the Creative Commons Attribution License (https://creativecommons.org/licenses/by/4.0/).

Comments for the author

Major concers

- Fig 1. The specificity of the pTAK1 antibodies is questionable. The authors point to one band in western blot that they define as non specific (I suppose based on the fact that it's still visible in the conditional KO, whereas the top band seems to disappear). However, over longer exposure some signal for pTAK1 is visible also on the "specific" band in the conditional KO, whereas no TAK1 seems to be detected in this sample. The authors should verify specificity of the antibody either in a total KO (as it's possible that some TAK1 is expressed by glial or endothelial cells in the brain) or at least in neurons where TAK1 can be knocked down.

- The experiments on the mouse models are very interesting, however it's unclear why the behaviour is performed only on APP/PS1 crossed with the TAK1 conditional KO (and no other experiments are performed on this model) whereas the majority of the characterization is made on the PS19 tau model crossed with the TAK1 conditional KO. I'm not questioning the validity of these experiments but I feel that a focus on one or the other model would make more sense. Or at least some behavioural experiments (Morris water maze and/or novel object recognition) should be performed on the PS19/TAK1cKO.

- After an initial characterization of the animal models, all the mechanistic experiments are performed in cell lines with the exception of a cell viability assay performed in cultured neurons. I feel like the activation of TAK1 in mature primary neurons upon glutamate+TNF treatment should be shown and in addition, the contribution of RIPK3 in this pathway should also be shown in primary neurons. This can be done for example by knocking down the protein and show resistance to toxicity induced by glutamate+TNF.

- The pathway is quite unclear to me. On one hand TAK1 activation mediated by calcium and TNF requires RIPK3 (Fig 5A), on the other hand RIPK3 activation is decreased upon TAK1 knockdown (Fig 5D). It is possible there is a feed loop mechanism at play but I think this should be demonstrated. Does complete absence of TAK1 completely block RIPK3 activation?
Linked to this, it is unclear in which step CamKIIb is involved in this pathway. Considering that HeLa cells seem not to express CamKIIb (Human protein atlas), it would seem that CamKIIb is not necessary nor sufficient for TAK1 activation (Fig 5A and 6A). Are the two pathways independent or does CamKIIbeta further activate RIPK3? Showing pRIPK3 levels in the experiments in Fig6 would partially answer this question.

Minor:

- Fig 4A, B. Please show representative images related to these graphs.
- Fig 5B. Most of these blots are oversaturated making it difficult to see the increase in phosphorylation, especially for MLKL. Please show a less saturated exposure and if the increase in MLKL is not obvious please show a quantification of this.
- Fig 5D. Given that the knockdown of TAK1 seems to reduce the total levels of RIPK3 too, the coIP levels of MLKL should be quantified as coIPed MLKL over IPed RIPK3
- Page 6, line 10-14, "Importantly, Nestin-Cre…". This paragraph is a bit confusing (see also the point about antibody specificity above). If the Tak1 signal is specific I would rather say that actually Tak1 is only or at least majorly expressed by neurons rather than glial cells as it seems to disappear in the nestin-cre KO.
- Supplementary fig 1. The blot for Tak1 in the cerebellar samples is not great, it would be nice to show another with clearer bands.
- Page 8, line 14-18, "Pathogenic stages…". This paragraph refers to an hypothesis for AD pathogenesis on which there is not full consensus in the field. I would state this clearly.
- Page 15, line 8, relative to Supplementary figure 5. "Indeed, TAK1 inhibitors…". Here it is stated that both 5ZOZ and Takinib block expression of inflammatory cytokines and chemokines, however in the figure Takinib appears to be effective only on IL1beta. Please make this clear.

© 2023. Published by The Company of Biologists under the terms of the Creative Commons Attribution License (https://creativecommons.org/licenses/by/4.0/).

First revision

Author response to reviewers' comments

Dear the reviewers:

We appreciate the reviewers' helpful comments in improving our manuscript. We have put our maximum efforts to address the reviewers' concerns. We apologize that the revision took longer than the standard revision period. It took longer mainly because the primary neuron culture experiments particularly with a new strain (*Ripk3-/-*) needed long time to obtain the sufficient mouse number. The major changes including new results are summarized below.

Major changes:
1. We have newly isolated and analyzed ***Ripk3-/-*** **primary neuron culture** (new Fig. 5F and supplementary Fig. S5B. We found that *Ripk3* deletion effectively rescues TNF and glutamate-induced neuronal loss. This supports our conclusion that compound stimulation of TNF and glutamate kills neurons through necroptosis.

2. We have demonstrated that co-stimulation of **TNF and glutamate activated TAK1 in primary neuron culture cells,** while TNF or glutamate alone were less effective in TAK1 activation (new Fig. 4C). This directly demonstrated that compound stimulation of TNF and glutamate activates TAK1 in neurons.

3. We have also examined whether necroptosis is induced in TNF and glutamate-treated primary neurons by analyzing **MLKL activation** (new Fig. 5E). This further support our conclusion that co-stimulation of TNF and glutamate elicits necroptosis.

4. We have added **a new model illustration** to summarize our findings (new Fig. 6D).

The list of new and revised figures are as follows. Fig. 4A (revised)
Fig. 4C (new)
Fig. 5A (revised, including quantification results) Fig. 5E (new)
Fig. 5F (new)
Fig. 6D (new summary illustration)
Supplementary Fig. S4 (new)
Supplementary Fig. S5B (new)

The point-by-point responses to the reviewers' comments are as follows. The reviewers' comments are indicated in *italic*. Finally, we note here that a new graduate student, Aoi Nakanishi, has contributed to the revision, and is listed as a co-author.

Reviewer 1:
1. In the current study, the authors express neuronal death observed in this study as necroptosis. By contrast, they mentioned "TAK1 mediates programmed cell death pathways including apoptosis, pyroptosis and necroptosis." in the introduction section. Exactly, although they found the phosphorylation of both MLKL and RIPK3, which are crucial in the induction of necroptosis, it is somewhat of an overestimation to characterize the cell death observed in the current study as necroptosis, based on these findings alone. It is necessary to improve.

Response:
We totally agree with the reviewer. It is important to demonstrate that neuronal loss is due to necroptosis. To determine whether necroptosis is the cause of TNF and glutamate-induced neuronal loss, we have newly analyzed *Ripk3-/-* primary neuron culture. We found that *Ripk3* deletion largely rescued TNF and glutamate-induced neuronal loss (new Fig. 5F and new supplementary Fig. S5B). This supports the conclusion that necroptosis is the major pathway of neuronal loss in TNF and glutamate co-stimulation.
We want to note here that our original statement, "TAK1 mediates programmed cell death pathways including apoptosis, pyroptosis and necroptosis" might be confusing, as TAK1 activation is associated with necroptosis while TAK1 inhibition promotes caspase 8 activation (apoptosis and

© 2023. Published by The Company of Biologists under the terms of the Creative Commons Attribution License (https://creativecommons.org/licenses/by/4.0/).

pyroptosis) (Morioka et al., 2014; Orning et al., 2018; Sarhan et al., 2018). We have removed the confusing statement on page 5.

2. the author used the one-way ANOVA with Tukey's multiple comparisons test in statistic analysis. In Fig. 4, the number of data is three, but in this case of this analysis, the number of data should be more than four.

Responses:
We agree with the reviewer. We have isolated additional primary neuron cultures from new embryos of 3 different mothers, and added data points (revised Fig. 4A).

3. It is necessary to mention why the concentration of TNF used differs between Fig. 4A and 4B.

Responses:
We have removed the original data using HT22 (neuron-like cell line), and added the results using a more physiologically relevant system, primary neuron culture (new Fig. 4C). The concentrations of TNF used were the same in Fig. 4A, B and C.

4. In Fig. 4C or 5, it is necessary to add quantitative evaluation of Western blots.

Responses:
We have added quantifications for Fig. 4C and Fig. 5A.

5. In Fig. 5B, the authors should examine whether treatment with either TNF or ionomycin alone induce the phosphorylation of MLKL and RIPK3.

Responses:
We have newly determined that co-stimulation of TNF and glutamate but not either single stimulation effectively activated MLKL (new Fig. 5E). While this new data did not exactly answer the reviewer's comment, we believe that the results using primary neurons instead of HeLa would be more valuable.

6. In Fig. 5D, deletion of TAK1 blocked the the phosphorylation of MLKL and RIPK3. These findings led to speculate that TAK1 might mediate activities of MLKL and RIPK3. By contrast, in Fig. 6, the overexpression of RIPK3 increased the phosphorylation of TAK1. Thus, the authors should discuss these pathways about the regulation of TAK-RIPK3.

7. In addition, treatment with ionomycin alone did not induce phosphorylation of TAK1, but co-treatment with TNF and ionomycin synergistically induced. Then, what mechanisms are involved in this regulation? It is necessary to examine and discuss more. I recommend to examine how the phosphorylation of CaMK Ⅱ changes under the treatment conditions described above.

8. I recommend to add a figure summarized the current findings.

Responses:
We appreciate the reviewer's suggestions. We have added the text discussing how TNF and CaMKII activate TAK1-RIPK3-MLKL (necroptosis) pathway (indicated in blue in Discussion). We have previously reported that i) overexpression of CaMKII activates TAK1 activity (Ishitani et al., 2003), ii) TNF-induced TAK1 activation is exacerbated by loss of TAK1 binding protein 2 (TAB2) that leads to RIPK3 activation (Morioka et al., 2014); and iii) in this context, activated RIPK3 further activates TAK1 (TAK1-RIPK3 feed-forward loop) (Morioka et al., 2014). Additionally, our unpublished results and the public proteomics data indicate a physical interaction between CaMKII and TAB2.
Taken together, we propose that calcium-CaMKII may modulate TAB2 resulting in elevated TNF-dependent TAK1 activation leading to necroptosis (see the schematic illustration in Fig. 6D). We aware of this statement needs more supportive results. We are currently working on this hypothesis and wish to report it in a future manuscript.

© 2023. Published by The Company of Biologists under the terms of the Creative Commons Attribution License (https://creativecommons.org/licenses/by/4.0/).

Reviewer 2

Major concerns

1. Fig 1. The specificity of the pTAK1 antibodies is questionable. The authors point to one band in western blot that they define as non specific (I suppose based on the fact that it's still visible in the conditional KO, whereas the top band seems to disappear). However, over longer exposure some signal for pTAK1 is visible also on the "specific" band in the conditional KO, whereas no TAK1 seems to be detected in this sample. The authors should verify specificity of the antibody either in a total KO (as it's possible that some TAK1 is expressed by glial or endothelial cells in the brain) or at least in neurons where TAK1 can be knocked down.

Responses:

We agree that the quality of pTAK1 antibody should be carefully evaluated. We have previously evaluated the specificity of this pTAK1 antibody (the same lot) using complete TAK1 catalytic activity null mutant cells (Omori et al., 2012). This pTAK1 antibody reacts with no non-specific proteins around the position of TAK1 but some weak non-specific bands appear at lower molecular weight positions in TAK1 catalytic activity null cell lysates [please see the figure for the reviewer's inspection below (Omori et al., 2012)]. We have modified the text to clarify the specificity of pTAK1 antibody in the figure legend of Fig. 1.

We also agree that microglia and endothelial cells, which do not express Nestin-Cre, may have activated TAK1, while the number of these cell types in the hippocampus are very small so that TAK1 protein was almost undetectable in Fig. 1A

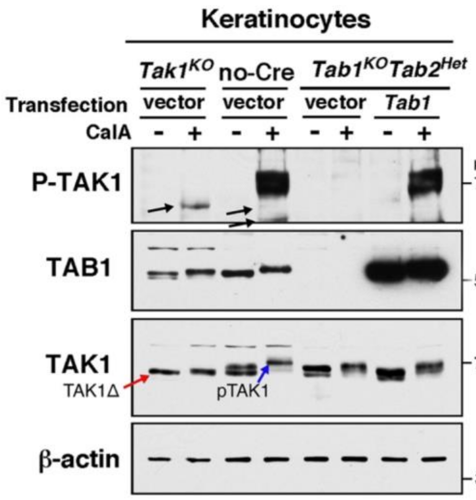

Figure for the reviewers' inspection

This is a figure from our publication (Omori et al., 2012). This result indicates that, with total KO of TAK1 catalytic activity, the pTAK1 antibody does not detect any non-specific bands around the position of TAK1. Please see the left 4 lanes. *Tak1* mutant (Tak1KO) or Tak1flox/flox (no-Cre) primary keratinocytes treated with and without a TAK1 activator, calyculin A (a phosphatase inhibitor) were analyzed by immunoblotting. In our TAK1 KO system, 38 amino acids including the ATP binding site are deleted by Cre (Sato et al., 2005), which produces a slightly smaller TAK1 protein (see the 3rd panel, red arrow) in cultured keratinocytes. This truncated TAK1 is unstable in the *in vivo* settings as shown in Fig. 1A. The ATP binding site deletion abolishes TAK1 catalytic activity. There is non-specific bands (black arrows), which are at lower positions but not close to the position of TAK1 protein. Note: Activation of TAK1 occurs by autophosphorylation and activated TAK1 further phosphorylates TAK1 at multiple sites that renders the TAK1 protein migration slower (blue arrow).

2. The experiments on the mouse models are very interesting, however it's unclear why the behaviour is performed only on APP/PS1 crossed with the TAK1 conditional KO (and no other experiments are performed on this model) whereas the majority of the characterization is made on the PS19 tau model crossed with the TAK1 conditional KO. I'm not questioning the validity of these experiments but I feel that a focus on one or the other model would make more sense. Or at least some behavioural experiments (Morris water maze and/or novel object recognition) should be performed on the PS19/TAK1cKO.

© 2023. Published by The Company of Biologists under the terms of the Creative Commons Attribution License (https://creativecommons.org/licenses/by/4.0/).

Responses:
We agree with the reviewer. We attempted the behavior experiments in the PS19 model mice, too. However, we found that PS19 mice have a phenotype of hindlimb paralysis starting from 6 months-12 months old. As their mobility is disturbed, reliable behavior results from PS19 mice were not obtained in the Morris water maze and the novel object assay. We have added this clarification in the text (page 7-8, indicated in blue).

3. After an initial characterization of the animal models, all the mechanistic experiments are performed in cell lines with the exception of a cell viability assay performed in cultured neurons. I feel like the activation of TAK1 in mature primary neurons upon glutamate+TNF treatment should be shown and in addition, the contribution of RIPK3 in this pathway should also be shown in primary neurons. This can be done for example by knocking down the protein and show resistance to toxicity induced by glutamate+TNF.

Responses:
We agree with the reviewer. We have examined whether TNF and glutamate activate TAK1 (new Fig. 4C) and MLKL (new Fig. 5E) in primary neuron culture. We have also used primary neuron culture from *Ripk3-/-* embryos and examined neuronal loss (new Fig. 5F). Our new results demonstrate that TNF and glutamate co-stimulation elicits necroptosis in primary neurons.

4. The pathway is quite unclear to me. On one hand TAK1 activation mediated by calcium and TNF requires RIPK3 (Fig 5A), on the other hand RIPK3 activation is decreased upon TAK1 knockdown (Fig 5D). It is possible there is a feed loop mechanism at play but I think this should be demonstrated. Does complete absence of TAK1 completely block RIPK3 activation?
Linked to this, it is unclear in which step CamKIIb is involved in this pathway. Considering that HeLa cells seem not to express CamKIIb (Human protein atlas), it would seem that CamKIIb is not necessary nor sufficient for TAK1 activation(Fig 5A and 6A). Are the two pathways independent or does CamKIIbeta further activate RIPK3? Showing pRIPK3 levels in the experiments in Fig6 would partially answer this question.

Responses:
<u>About the overall pathway</u>
We appreciate the reviewer's comment. We have added a discussion of how calcium- CaMKII facilitate TNF-induced TAK1 activation. The proposed pathway is summarized in the new schematic illustration (Fig. 6D).
<u>CaMKII expression in HeLa cells</u>
Other CaMKII isoforms such as delta and gamma are expressed in HeLa cells (Human protein atlas). These CaMKIIs are functionally similar to beta.

Minor:
-Fig 4A, B. Please show representative images related to these graphs.-Fig 5B. Most of these blots are oversaturated making it difficult to see the increase in phosphorylation, especially for MLKL. Please show a less saturated exposure and if the increase in MLKL is not obvious please show a quantification of this.-Fig 5D. Given that the knockdown of TAK1 seems to reduce the total levels of RIPK3 too, the coIP levels of MLKL should be quantified as coIPed MLKL over IPed RIPK3

Responses:
We have tried to quantify the immunoblotting as much as possible (new Fig. 4C and revised Fig. 5A). However, we regrettably say that we were not able to quantify all immunoblotting results.

-Page 6, line 10-14, "Importantly, Nestin-Cre…". This paragraph is a bit confusing (see also the point about antibody specificity above). If the Tak1 signal is specific I would rather say that actually Tak1 is only or at least majorly expressed by neurons rather than glial cells as it seems to disappear in the nestin-cre KO.

Responses:
We appreciate the reviewer's comments. The original sentence was confusing. In this sentence, we wanted to state that the observed TAK1 activation was not due to TAK1 activation in microglia or endothelial cells, which are known to express TAK1. As the reviewer pointed out that majority

© 2023. Published by The Company of Biologists under the terms of the Creative Commons Attribution License (https://creativecommons.org/licenses/by/4.0/).

of hippocampal cells are neurons.
We have revised the text in page 6 (indicated in blue).

-Supplementary fig 1. The blot for Tak1 in the cerebellar samples is not great, it would be nice to show another with clearer bands.

Responses:
Regrettably, we do not have enough amounts of samples to re-try Western blottings.

-Page 8, line 14-18, "Pathogenic stages…". This paragraph refers to an hypothesis for AD pathogenesis on which there is not full consensus in the field. I would state this clearly.

Responses:
We appreciate the reviewer's comments. We have revised the text to indicate that these are the list of AD pathological events but not the order of AD pathogenesis in page 9 (indicated in blue).

-Page 15, line 8, relative to Supplementary figure 5. "Indeed, TAK1 inhibitors…". Here it is stated that both 5ZOZ and Takinib block expression of inflammatory cytokines and chemokines, however in the figure Takinib appears to be effective only on IL1beta. Please make this clear.

Responses:
We appreciate the reviewer's comments. We have revised the text in page 16 (indicated in blue).

We hope that our responses would address most of reviewers' concerns. We appreciate your consideration.

Best regards,

Kazuhito Sai
Jun Ninomiya-Tsuji

Dept. of Biological Sciences
North Carolina State University

References:

Ishitani, T., Kishida, S., Hyodo-Miura, J., Ueno, N., Yasuda, J., Waterman, M., Shibuya, H., Moon, R. T., Ninomiya-Tsuji, J. and Matsumoto, K. (2003). The TAK1-NLK mitogen- activated protein kinase cascade functions in the Wnt-5a/Ca(2+) pathway to antagonize Wnt/beta-catenin signaling. *Mol. Cell. Biol.* **23**, 131-139.

Morioka, S., Broglie, P., Omori, E., Ikeda, Y., Takaesu, G., Matsumoto, K. and Ninomiya-Tsuji, J. (2014). TAK1 kinase switches cell fate from apoptosis to necrosis following TNF stimulation. *J. Cell Biol.* **204**, 607-623.

Omori, E., Inagaki, M., Mishina, Y., Matsumoto, K. and Ninomiya-Tsuji, J. (2012). Epithelial transforming growth factor beta-activated kinase 1 (TAK1) is activated through two independent mechanisms and regulates reactive oxygen species. *Proc. Natl. Acad. Sci. U. S. A.* **109**, 3365-3370.

Orning, P., Weng, D., Starheim, K., Ratner, D., Best, Z., Lee, B., Brooks, A., Xia, S., Wu, H., Kelliher, M. A. et al. (2018). Pathogen blockade of TAK1 triggers caspase-8-dependent cleavage of gasdermin D and cell death. *Science* **362**, 1064-1069.

Sarhan, J., Liu, B. C., Muendlein, H. I., Li, P., Nilson, R., Tang, A. Y., Rongvaux, A., Bunnell, S. C., Shao, F., Green, D. R. et al. (2018). Caspase-8 induces cleavage of gasdermin D to elicit pyroptosis during Yersinia infection. *Proc. Natl. Acad. Sci. U. S. A.* **115**, E10888- E10897.

© 2023. Published by The Company of Biologists under the terms of the Creative Commons Attribution License (https://creativecommons.org/licenses/by/4.0/).

Sato, S., Sanjo, H., Takeda, K., Ninomiya-Tsuji, J., Yamamoto, M., Kawai, T., Matsumoto, K., Takeuchi, O. and Akira, S. (2005). Essential function for the kinase TAK1 in innate and adaptive immune responses. *Nat. Immunol.* **6**, 1087-1095.

Second decision letter

MS ID#: JOCES/2022/260102

MS TITLE: Aberrantly activated TAK1 links neuroinflammation and neuronal loss in Alzheimer's disease mouse models

AUTHORS: Kazuhito Sai, Aoi Nakanishi, Kimberly M Scofield, Debra A Tokarz, Keith E Linder, Todd J Cohen, and Jun Ninomiya-Tsuji
ARTICLE TYPE: Research Article

We have now reached a decision on the above manuscript.

To see the reviewers' reports and a copy of this decision letter, please go to: https://submit-jcs.biologists.org and click on the 'Manuscripts with Decisions' queue in the Author Area. (Corresponding author only has access to reviews.)

As you will see, the last reviewer gave favourable reports but raised some critical points that will require amendments to your manuscript. I hope that you will be able to carry these out because I would like to be able to accept your paper

Please ensure that you clearly highlight all changes made in the revised manuscript. Please avoid using 'Tracked changes' in Word files as these are lost in PDF conversion.

I should be grateful if you would also provide a point-by-point response detailing how you have dealt with the points raised by the reviewers in the 'Response to Reviewers' box. Please attend to all of the reviewers' comments. If you do not agree with any of their criticisms or suggestions please explain clearly why this is so.

Reviewer 2

Advance summary and potential significance to field

The paper from Sai and colleagues describes an interesting link between the inflammation related mitogen activated protein kinase kinase kinase TAK1 and neuronal cell death, with a focus on Alzheimer's disease.
Interestingly, TAK1 is hyperactivated in aged mice and in APP/PS1 and PS19 (tau) models, and the neuron-specific depletion of TAK1 is able to revert well-known deficits present in these models such as spatial memory in APP/PS1 and neuronal loss in PS19. In separate experiments they show that TAK1 can be hyperactivated by co application of glutamate or Ionomycin and TNF in cell lines, a treatment that induces neuronal death. TAK1 activation appears to be dependent on RIPK3/MLKL and on CamKIIb.
The results are of good quality and interesting for the field.

Comments for the author

I appreciate the efforts from the authors to reply to the comments and the manuscript has certainly improved from the previous version. However, there are still some points which I feel need to be addressed better.
Most importantly, I feel like the first point of the previous revision regarding the specificity of the pTak1 antibody has not fully been answered and it still remains unclear how there could be pTAK1 signal in the absence of TAK1 (as it is shown in the rightmost lane in figure 1A). In addition to the

© 2023. Published by The Company of Biologists under the terms of the Creative Commons Attribution License (https://creativecommons.org/licenses/by/4.0/).

evidence from previous work provided, the authors have to show a longer exposure of the TAK1 blot, to highlight whether there is low levels of TAK1 in that sample.
In addition, the 4th point of the previous revision is also gone un-replied- Can the author show phosphorylated RIPK3 levels in the experiment in figure 6A? This would help to understand the contribution of CamKII to the pathway.

Second revision

Author response to reviewers' comments

The point-by-point responses are as follows.

1) Comment: Most importantly, I feel like the first point of the previous revision regarding the specificity of the pTak1 antibody has not fully been answered and it still remains unclear how there could be pTAK1 signal in the absence of TAK1 (as it is shown in the rightmost lane in figure 1A). In addition to the evidence from previous work provided, the authors have to show a longer exposure of the TAK1 blot, to highlight whether there is low levels of TAK1 in that sample.

Response:

We agree the reviewer's point. We have added a new Western blotting using the same samples (revised Fig. 1A, bottom panel). As we did not have a longer exposure of the TAK1 Western blotting shown the third pane, we used a different membrane loaded with the same samples. There is a very weak but clearly visible intact TAK1 band in the Tak1CNSKO lane. We have also revised the main text and the figure legend accordingly (indicated in blue).

2) Comment: In addition, the 4th point of the previous revision is also gone un-replied- Can the author show phosphorylated RIPK3 levels in the experiment in figure 6A? This would help to understand the contribution of CamKII to the pathway.

Response:

We agree that showing RIPK3 activation is informative. However, we could not find an effective antibody to specifically detect phosphorylated human RIPK3. Instead of re-blotting with a phospho-specific antibody , we have added a shorter exposure of HA-RIPK3 Western blotting (revised Fig. 6A, bottom panel). This suggests that overexpression of active CaMKII alone does not activate RIPK3. This supports our conclusion summarized in Fig. 6 D. We have also revised the main text accordingly (indicated in blue).

Thank you for your consideration.

Third decision letter

MS ID#: JOCES/2022/260102

MS TITLE: Aberrantly activated TAK1 links neuroinflammation and neuronal loss in Alzheimer's disease mouse models

AUTHORS: Kazuhito Sai, Aoi Nakanishi, Kimberly M Scofield, Debra A Tokarz, Keith E Linder, Todd J Cohen, and Jun Ninomiya-Tsuji
ARTICLE TYPE: Research Article

I am happy to tell you that your manuscript has been accepted for publication in Journal of Cell Science, pending standard ethics checks.

© 2023. Published by The Company of Biologists under the terms of the Creative Commons Attribution License (https://creativecommons.org/licenses/by/4.0/).
